# Paired Box 9 (PAX9), the RNA polymerase II transcription factor, regulates human ribosome biogenesis and craniofacial development

**DOI:** 10.1371/journal.pgen.1008967

**Published:** 2020-08-19

**Authors:** Katherine I. Farley-Barnes, Engin Deniz, Maya M. Overton, Mustafa K. Khokha, Susan J. Baserga

**Affiliations:** 1 Department of Molecular Biophysics & Biochemistry, Yale University School of Medicine, New Haven, Connecticut, United States of America; 2 Pediatric Genomics Discovery Program, Department of Pediatrics, Yale University School of Medicine, New Haven, Connecticut, United States of America; 3 Department of Genetics, Yale University School of Medicine, New Haven, Connecticut, United States of America; 4 Department of Therapeutic Radiology, Yale University School of Medicine, New Haven, Connecticut, United States of America; Stowers Institute for Medical Research, UNITED STATES

## Abstract

Dysregulation of ribosome production can lead to a number of developmental disorders called ribosomopathies. Despite the ubiquitous requirement for these cellular machines used in protein synthesis, ribosomopathies manifest in a tissue-specific manner, with many affecting the development of the face. Here we reveal yet another connection between craniofacial development and making ribosomes through the protein Paired Box 9 (PAX9). PAX9 functions as an RNA Polymerase II transcription factor to regulate the expression of proteins required for craniofacial and tooth development in humans. We now expand this function of PAX9 by demonstrating that PAX9 acts outside of the cell nucleolus to regulate the levels of proteins critical for building the small subunit of the ribosome. This function of PAX9 is conserved to the organism *Xenopus tropicalis*, an established model for human ribosomopathies. Depletion of *pax9* leads to craniofacial defects due to abnormalities in neural crest development, a result consistent with that found for depletion of other ribosome biogenesis factors. This work highlights an unexpected layer of how the making of ribosomes is regulated in human cells and during embryonic development.

## Author summary

We are only beginning to understand the complex process of making human ribosomes, the cellular machines critical for all protein synthesis. In humans, making a ribosome requires hundreds of regulatory factors to ensure proper cellular growth and development. Dysregulation of this process can lead to a number of tissue specific disorders, termed ribosomopathies. Here, we have discovered a new role for the protein Paired Box 9 (PAX9) in making human ribosomes. While PAX9 has traditionally been known to play a role in regulating the levels of proteins required for craniofacial and tooth development in humans, we expand this function of PAX9 by showing that PAX9 acts outside of the cell nucleolus to regulate the levels of proteins critical for building the small subunit of the ribosome. In addition, we show that this function is conserved to the model organism, *Xenopus tropicalis*. This link between PAX9’s role in craniofacial development and in ribosome biogenesis may lead to new insights into the pathogenesis of these *PAX9* mutations in humans.

## Introduction

When ribosome biogenesis is genetically disrupted in humans, a number of surprisingly tissue-specific disorders called ribosomopathies arise. For example, genetic disruption of the *TCOF1*, *POLR1C*, or *POLR1D* genes in the ribosomopathy, Treacher Collins syndrome (TCS, OMIM 154500), results in reduced pre-ribosomal RNA (pre-rRNA) transcription [1–4]. Interestingly, while these mutations each affect the global process of pre-rRNA transcription, patients have specific defects in craniofacial development. TCS patients present with hypoplasia of the facial bones, micrognathia with or without cleft palate, narrowing of the ear canal, and bilateral conductive hearing loss [5–7]. Modeling the disease in mice has shown that this tissue specificity arises from differential tissue susceptibility to p53 levels [8]. p53 levels are stabilized upon disruptions in ribosome biogenesis when free ribosomal proteins bind to MDM2, the E3 ligase for p53 [9, 10]. This stabilization of p53 leads to apoptosis of the developing neural crest cells, ultimately resulting in the mandibulofacial dysostosis of TCS.

TCS is not the only ribosomopathy to affect craniofacial development. For example, Diamond Blackfan anemia (DBA, OMIM 105650) is characterized by anemia, low reticulocyte count, and elevated erythrocyte adenosine deaminase activity [11, 12]. However, DBA patients often also have craniofacial anomalies and cleft palate (reviewed in [13]). Additionally, the ribosomopathy Acrofacial dysostosis, Cincinnati type, (OMIM 616462) is caused by mutations in *POLR1A* that inhibit pre-rRNA transcription, resulting in craniofacial defects [14]. A more precise understanding of the role of the factors involved in human ribosome biogenesis can therefore shed light on the molecular mechanisms underlying aberrant craniofacial development.

**Fig 1:**
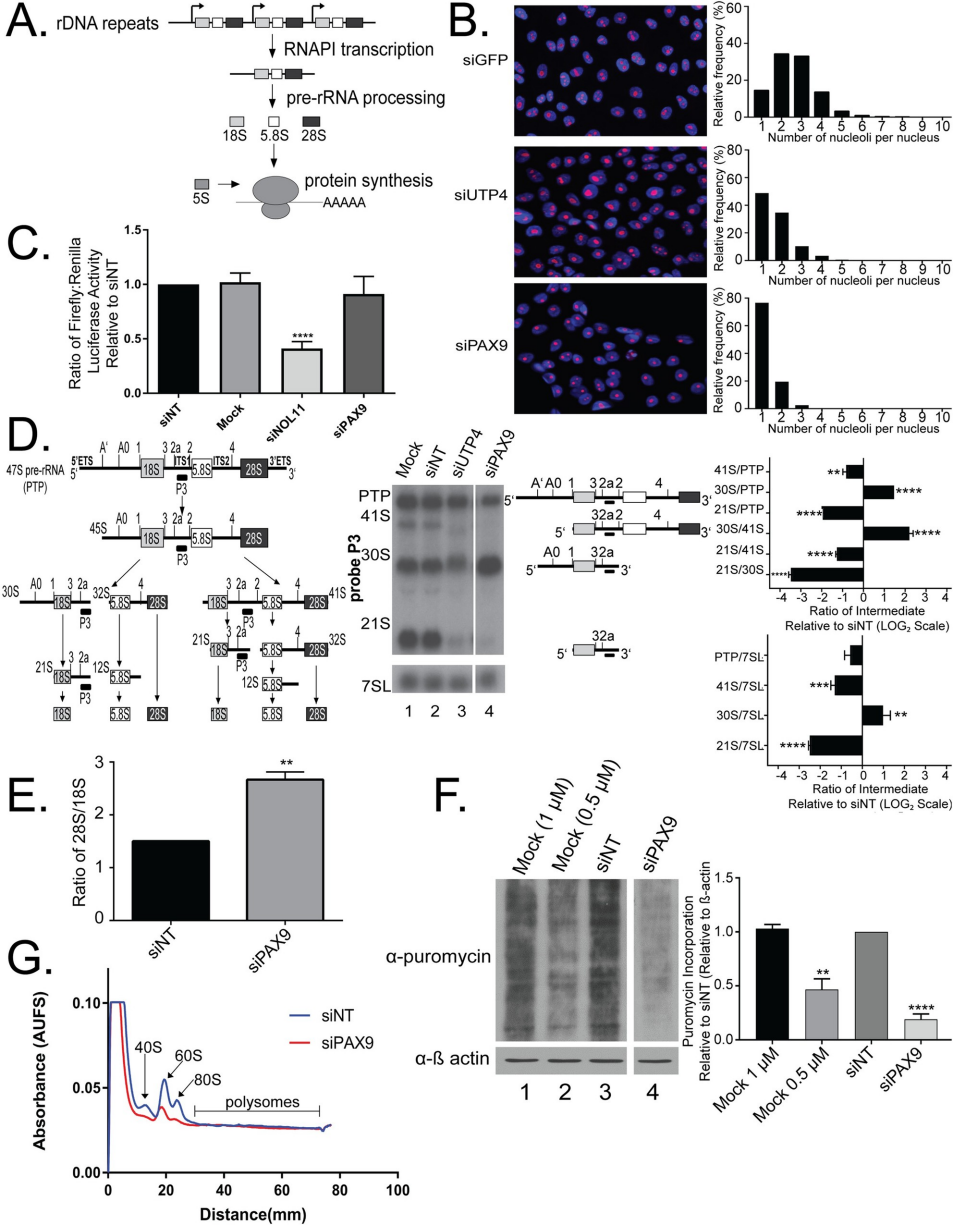
PAX9 is required for human ribosome biogenesis. (A) Ribosome biogenesis at a glance. The tandemly repeated ribosomal DNA (rDNA) is transcribed into the 47S polycistronic pre-ribosomal RNA (pre-rRNA) by RNA Polymerase I (RNAPI). This 47S pre-rRNA is processed through multiple steps to form the mature 18S, 5.8S, and 28S rRNAs which are incorporated into the small and large subunits of the ribosome, along with the 5S rRNA and 80 ribosomal proteins. Ribosomes perform cytoplasmic cellular protein synthesis through the translation of mRNAs. (B) PAX9 depletion reduces nucleolar number from 2–3 to only 1 in MCF10A cells. Left panel: Nuclei stained in Hoechst are shown in blue. Nucleoli are shown in pink and stained with anti-fibrillarin antibody as in [34]. siGFP (top) was used as a negative control (2–3 nucleoli/nucleus) and siUTP4 (middle) was used as a positive control (1 nucleolus/nucleus). siPAX9 is shown at the bottom. Right panel: Quantitation of the number of nucleoli per nucleus for siGFP (top), siUTP4 (middle), or siPAX9 (bottom). (C) PAX9 is not required for RNAPI transcription in MCF10A cells. A dual-luciferase reporter assay was used to quantify luminescence after siRNA depletion of PAX9. The 2 plasmids are pHrD-IRES-Luc (firefly) to report RNAPI transcription and a *Renilla* transfection control as in [34]. The ratio of firefly to *Renilla* luciferase was normalized to the siNT control. N = 4. siNOL11 was used as a positive control [38]. Data were analyzed by Student’s t test using GraphPad Prism. **** p ≤ 0.0001. (D) PAX9 is required for pre-18S rRNA processing in MCF10A cells. Left: Schematic of pre-rRNA processing steps in human cells. Intermediates detected by probe P3 are indicated with a black box below. Center: Northern blot with probe P3. A probe for the 7SL RNA was used as a loading control. Intermediates detected by probe P3 are shown to the right of the northern blot. Negative controls were mock (no siRNA) and siNT (non-targeting). siUTP4 was used as a positive control [38]. Right: Quantitation by RAMP [40] of probe P3 (upper) and 7SL (lower) northern blots. Graph is mean ± SEM. N = 3. Data were analyzed by 2-way ANOVA using GraphPad Prism. **** p ≤ 0.0001, *** p ≤ 0.001, ** p ≤ 0.01, and * p ≤ 0.05. PTP indicates the 47S, 45S, and 43S processing intermediates. (E) PAX9 siRNA depletion in MCF10A cells results in an increased ratio of 28S/18S by Agilent BioAnalyzer. Significance was calculated by Student’s t test in GraphPad Prism where ** p ≤ 0.01. (F) PAX9 siRNA depletion in MCF10A cells results in decreased global protein synthesis as assessed by the puromycin incorporation assay [41]. A representative western blot using an anti-puromycin antibody with a β actin loading control is shown to the left. Protein was harvested after knockdown for 72 hours using the indicated siRNAs. Mock indicates no siRNA and Mock 0.5 μM indicates no siRNA and half the concentration of puromycin. siNT (non-targeting) was used a positive control. Quantitation of 3 replicates using cells of different passage numbers is shown to the right. Significance was calculated by One-way ANOVA in GraphPad Prism where **** p ≤ 0.0001 and *** p ≤ 0.001. (G) PAX9 depletion in MCF10A cells results in decreased 40S, 60S, and 80S ribosome subunit levels. Representative polysome profile of MCF10A cells depleted using siRNAs targeting either PAX9 (red) or a non-targeting (siNT, blue) control. Equal amounts of protein were loaded on each gradient. This experiment was performed 3 times using cells of different passage numbers.

The process of making the cellular machines required for protein synthesis, called ribosome biogenesis, includes a large number of factors that work together to control cell growth and development. In humans, these proteins are still being defined. Previous studies have shown that ribosome biogenesis begins with the transcription of the tandemly repeated ribosomal DNA (rDNA) into the 47S polycistronic pre-rRNA (Fig 1A). The pre-rRNA is further modified and processed to create the mature 18S, 5.8S, and 28S rRNAs. These rRNAs are incorporated, along with the 5S rRNA and ribosomal proteins, into the small (SSU) and large (LSU) subunits of the ribosome. The 47S pre-rRNA is transcribed by RNA polymerase I (RNAPI), while the 5S rRNA is transcribed by RNA polymerase III (RNAPIII). Various assembly factors and the 80 ribosomal proteins are transcribed by RNA polymerase II (RNAPII). In addition to requiring all 3 RNA polymerases, synthesis and assembly of functional ribosomes integrates a number of cellular signaling pathways to ensure proper regulation of this essential process in response to stimuli.

The complex development of the face is controlled by a number of proteins, including several RNAPII transcription factors such as Paired Box 9 (PAX9). PAX9 belongs to a family of transcription factors that play key roles in organogenesis and neural crest cell development by controlling gene expression [15, 16]. In humans, mutations in PAX9 cause tooth agenesis as well as hair loss ([17–24] and reviewed in [25]). Indeed, PAX9 mutations are the most prevalent mutation in patients with nonsyndromic tooth agenesis (including oligodontia; [26]). Additionally, mice homozygous for a partial deletion of Pax9 (likely null) have craniofacial malformations including cleft palate, skeletal abnormalities, and arrested tooth development and die a few hours after birth [27]. Pax9^-/-^ mice also exhibit a range of cardiac malformations [28], another commonly impacted tissue in ribosomopathies [29]. While some research has been done to identify the signaling pathways regulated by PAX9, attempts to correct the developmental defects have been only partially successful [30–33]. Therefore, further studies are needed to identify all factors regulated by PAX9 in order to understand PAX9’s role in craniofacial development.

Our work ties together PAX9 and ribosome biogenesis, filling in some gaps in our knowledge of the many cell growth and signaling pathways influenced by PAX9 depletion. We originally identified PAX9 as a potential regulator of ribosome biogenesis in an siRNA screen for proteins required to maintain nucleolar number [34]. Probing PAX9’s specific role in making ribosomes in human tissue culture cells, we discovered that PAX9 is required both for the pre-rRNA processing that produces the small subunit 18S rRNA and for global protein synthesis. We employed the genome-wide transcriptomics analysis, RNA-seq, to define PAX9 dependent mRNAs necessary for making ribosomes. Several of the differentially expressed mRNAs, including several ribosomal proteins, were further examined to pinpoint roles for these proteins in pre-rRNA processing and global protein synthesis. Finally, given an established role for PAX9 in human craniofacial development, we sought to model this disease in *Xenopus tropicalis* (*X*. *tropicalis*) embryos, an established model of human ribosomopathies. Depletion of Pax9 in *X*. *tropicalis* did indeed alter craniofacial patterning as well as neural crest development, a migratory cell population that plays a major role in establishing craniofacial structure. *X*. *tropicalis* embryos depleted of Pax9 also show defective pre-rRNA processing. These results shed light on the plethora of factors whose expression is regulated by PAX9, and open the door to likely connections between PAX9’s role in craniofacial development and human ribosome biogenesis.

## Results

### PAX9 depletion disrupts small subunit ribosome biogenesis

Previously, we performed an siRNA screen for new regulators of nucleolar number in human MCF10A cells [34]. We identified PAX9 by this screening approach as a protein that, when depleted, reduced the number of nucleoli per nucleus from 2–3 to only 1 (Fig 1B). As in [34], cells were fixed after 72 hours of knockdown using pools of siRNAs targeting siGFP as a negative control, siUTP4 as a positive control, or siPAX9. This transient knockdown approach successfully depleted PAX9 (S1A and S1B Fig). The MCF10A cells were then stained with an antibody to fibrillarin (FBL), a nucleolar protein, to detect nucleoli and with HOECHST, to detect nuclei. A CellProfiler [35] pipeline was used to quantify the number of nucleoli per cell nucleus, which shifted from 2–3 in siGFP control cells to only 1 in siPAX9 and siUTP4-treated cells. We had previously used oligonucleotide deconvolution to additionally confirm that PAX9 depletion leads to reductions in nucleolar number [34]. Using this approach, individual depletion of PAX9 using 3 of the 4 siRNAs from the original PAX9 pool reduced the number of nucleoli from 2–3 to only 1 per cell nucleus [36, 37]. As the siRNA screen served as a phenotypic readout of nucleolar function [34], we hypothesized that PAX9 plays a role in human ribosome biogenesis through its function as an RNAPII transcription factor.

We sought to investigate the extent to which PAX9 depletion affects human ribosome biogenesis using a panel of 3 assays. The first assay probes PAX9’s role in RNAPI transcription using a dual-luciferase reporter system previously published by our laboratory and others [34, 38, 39]. As PAX9 is a known RNAPII transcription factor, it was relevant to test it for a possible additional role in RNAPI transcription. In this reporter assay, the ratio of firefly luciferase, which is under the control of the rDNA promoter and measures RNAPI transcription, was quantified relative to a *Renilla* luciferase transfection control. Relative to a non-targeting control siRNA (siNT), siRNAs targeting PAX9 had no significant effect on RNAPI transcription levels after 72 hours of knockdown in MCF10A cells (Fig 1C). Mock (no siRNA) and siNOL11 were used as negative and positive controls, respectively. PAX9 is therefore not required for RNAPI transcription in MCF10A cells.

Northern blotting was used to define PAX9’s role in pre-rRNA processing. In humans, pre-rRNA processing occurs via a number of different pathways (Fig 1D, left) and requires a number of trans-acting factors. We therefore employed 6 different probes to pinpoint any pre-rRNA processing defects occurring after PAX9 depletion in MCF10A cells (S2A Fig). After 72 hours of siRNA knockdown, PAX9 depletion resulted in a significant increase in the 30S pre-rRNA intermediate as well as a decrease in the levels of its 21S processing product, relative to the non-targeting siRNA (siNT, Fig 1D, middle and right, and S2 Fig). Additionally, 41S levels were decreased relative to the primary processing transcript (47S) plus the 45S and 43S processing intermediates, herein termed the primary transcript plus, or PTP (Fig 1D, middle and right and S2 Fig). Quantitation of the ratios of each intermediate relative to its precursor in the processing pathway by Ratio Analysis of Multiple Precursors (RAMP; [40]) confirmed the statistical significance of these results (Fig 1D, right, and S2 Fig). These effects were also significant relative to a 7SL loading control (Fig 1D, right, and S2 Fig). Because the 30S and 21S intermediates are both precursors to the 18S rRNA, PAX9 is required for SSU biogenesis. The same pre-rRNA processing defect was also detected in human embryonic kidney (HEK293FT) and colon carcinoma (RKO) cells depleted of PAX9, indicating that PAX9’s role in ribosome biogenesis is conserved among diverse human cell lines (S1 Fig).

Because the pre-rRNA processing defects indicate aberrant SSU biogenesis, we sought to determine the extent to which PAX9 depletion affects the production of the mature 18S rRNA. Agilent BioAnalyzer quantitation shows an increase in the ratio of 28S to 18S (Fig 1E). Combined with the northern blot results indicating defects in the processing of the precursors to the 18S rRNA, this result is consistent with a predicted reduction in 18S rRNA levels. Taken together, these results argue that PAX9 is required likely indirectly for the biogenesis of the small subunit of the ribosome, which contains the 18S rRNA.

We also utilized a puromycin incorporation assay to test the extent to which PAX9 depletion alters the final product of ribosome biogenesis: global cellular translation (Fig 1F). Cells with or without PAX9 depletion are treated with a low dose (1 μM) of puromycin, which is incorporated into all nascent peptides produced during a 1 hour pulse [41]. Western blotting for incorporated puromycin shows decreased protein synthesis after 72 hours of PAX9 depletion, relative to a non-targeting siRNA control (siNT) (Fig 1F). Mock (1 μM puromycin with no siRNA) and Mock at a half-dose of puromycin (0.5 μM) were used as negative controls (Fig 1F). These results confirm that PAX9 depletion leads to reduced protein synthesis, consistent with a role for PAX9 in SSU biogenesis.

Because the above assays indicated a role for PAX9 in small subunit pre-rRNA processing and global protein synthesis, we also tested the extent to which PAX9 depletion is required for ribosomal subunit biogenesis and assembly. Using polysome profiling, we determined that PAX9 depletion does result in significantly decreased SSU (40S) levels in MCF10A cells (Fig 1G), consistent with the reduction in 18S rRNA levels seen in Fig 1E. Additionally, 60S and 80S polysome fractions also showed significant decreases after PAX9 knockdown (Fig 1G). Interestingly, both here and in previous experiments, MCF10A cells do not demonstrate robust polysome fractions using this technique [42]. Regardless, we conclude that PAX9 depletion results in decreased SSU biogenesis that impacts the assembly and function of the ribosome.

As disruptions in ribosome biogenesis result in interruptions in the cell cycle [43–48], we also examined the extent to which PAX9 depletion changed the distribution of MCF10A cells within the cell cycle using flow cytometry (S3 Fig). Relative to a siNT control, depletion of PAX9 for 72 hours resulted in a minor increase in the proportion of cells in G1 phase of the cell cycle, but this was not statistically significant (S3 Fig). siRNAs targeting the ribosome biogenesis factor NOL11 were used as a positive control, and depletion of this protein resulted in an increase in the proportion of cells in G2 (S3 Fig), consistent with previous findings [49]. In all, these assays allowed us to conclude that PAX9 regulates human ribosome biogenesis.

### RNA-seq analysis upon PAX9 depletion reveals decreased levels of nucleolar mRNAs responsible for small subunit maturation

**Fig 2:**
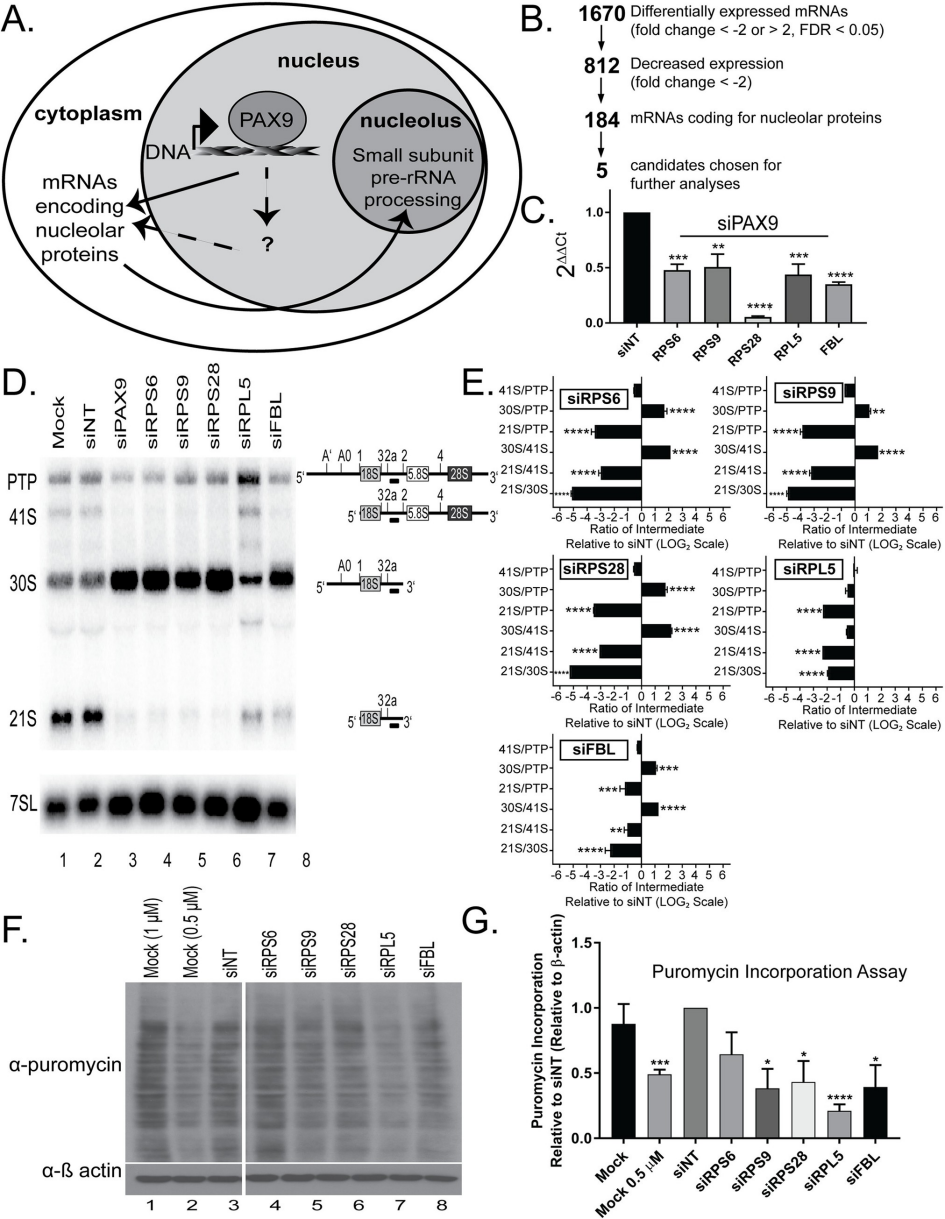
RNA-seq transcriptomics analysis in human tissue culture cells reveals changes in the expression levels of over 100 nucleolar mRNAs after PAX9 knockdown. (A) Schematic of how PAX9 would act as a RNAPII transcription factor to drive the levels of mRNAs required for making the small subunit (SSU) of the ribosome. In the cell nucleus, PAX9 binds to DNA to affect the transcription of mRNAs that either encode nucleolar proteins (direct; solid arrow) or to transcribe mRNAs that affect the levels of mRNAs encoding nucleolar proteins (indirect; dotted arrows). The resulting mRNAs are translated in the cytoplasm into proteins that function in SSU pre-rRNA processing in the nucleolus. (B) RNA-seq analysis after PAX9 siRNA depletion in MCF10A cells reveals decreased levels of mRNAs encoding 184 nucleolar proteins. Relative to a non-targeting siRNA control (siNT), PAX9 depletion resulted in differential expression of 1670 mRNAs (fold change ≤ -2 or > 2 and FDR ≤ 0.05). Of these, 812 mRNAs had a decreased fold change (≤ -2) and 184 of those mRNAs code for proteins designated as nucleolar in at least one of three databases [50–52]. Of the 184 mRNAs whose levels were decreased and that also code for nucleolar proteins, 5 were chosen as candidates for follow-up studies. (C) qRT-PCR confirms reduced mRNA levels of the 5 RNA-seq candidates after PAX9 siRNA knockdown in MCF10A cells. After depletion using siRNAs targeting either PAX9 or a non-targeting control siRNA (siNT), the levels of the indicated 5 mRNAs were quantified by qRT-PCR using primers to each target gene relative to a 7SL control and siNT. Data are shown as mean ± SEM. Three replicates using cells of different passage numbers, with 3 technical replicates each, were performed. Significance was calculated by One-way ANOVA using GraphPad Prism where **** p ≤ 0.0001. (D) Depletion of 4 of the 5 candidate mRNAs (RPS6/eS6, RPS9/uS4, RPS28/eS28, and FBL) individually results in the same pre-rRNA processing defect as PAX9 siRNA depletion in MCF10A cells. Representative northern blot after knockdown of the indicated siRNAs using probe P3. A probe for the 7SL RNA was used as a loading control. Pre-rRNA processing intermediates detected by probe P3 are shown to the right of the northern blot. PTP indicates the 47S, 45S, and 43S pre-rRNA processing intermediates. (E) Quantitation of northern blots using probe P3 as shown in (Fig 2D) using RAMP [40]. Graph is mean ± SEM. N = 3. Data were analyzed using 2-way ANOVA in GraphPad Prism where **** p ≤ 0.0001, *** p ≤ 0.001, and ** p ≤ 0.01. Quantitation relative to the 7SL loading control is shown in S5 Fig. (F) siRNA depletion of 4/5 RNA-seq candidates in MCF10A cells results in decreased global protein synthesis. After 72 hours of knockdown with the indicated siRNAs, MCF10A cells were pulsed with puromycin for 1 hour and protein was harvested. Western blotting with an anti-puromycin antibody as well as a β actin loading control was carried out (representative western blots shown to the left). Mock, mock at half the concentration of puromycin (0.5 μM), and siNT (non-targeting siRNAs) were used as negative controls. (G) Quantitation of three replicates using MCF10A cells of different passage numbers of the puromycin incorporation assays following depletion with the indicated siRNAs relative to the siNT and β actin loading controls is shown as mean ± SEM. N = 3. Significance was calculated by Student’s t-test using GraphPad Prism where **** p ≤ 0.0001, *** p ≤ 0.001, and * p ≤ 0.05.

As PAX9 is a known RNAPII transcription factor, we hypothesized that PAX9 works indirectly to modulate SSU biogenesis (Fig 2A). This is consistent with a nuclear but not nucleolar localization of PAX9 in 3 existing databases [50–52]. PAX9 may act directly as a transcription factor for nucleolar proteins, or indirectly for proteins that affect the expression or function of nucleolar proteins. To test the hypothesis that PAX9 affects nucleolar protein expression through its function as a RNAPII transcription factor, we used RNA-seq in MCF10A cells to define the set of mRNAs that were differentially expressed after PAX9 depletion. Relative to a non-targeting control siRNA (siNT), PAX9 depletion resulted in the differential expression of over 1,600 mRNAs (fold change ≥ 2 or ≤ -2, q ≤ 0.05) (Fig 2B and S1 Table). Approximately half (812) of these were reduced in their levels, consistent with the hypothesis that PAX9 acts as a transcription factor to drive their expression.

When considered as a whole, the RNA-seq dataset is enriched for several pathways known to be regulated by PAX9 (S4A Fig). Ingenuity Pathway Analysis [53] of the 1670 differentially expressed mRNAs reveals enrichment of both Wnt/Ca2+ signaling (differential expression of 10 pathway members, p = 1.04 x 10^−2^) (S4B Fig), and Wnt/β-catenin signaling (differential expression of 19 pathway members, p = 2.79 x 10^−2^) (S4C Fig). In cells depleted of PAX9, expression levels are increased for many of the mRNAs in the Wnt signaling pathway. This is consistent with previous results suggesting a role for PAX9 in the negative regulation of Wnt signaling [30, 31, 33].

As an additional validation of the RNA-seq dataset, we determined that the genome sequences 1 kb upstream of the start sites of the 1670 differentially expressed mRNAs contain multiple potential PAX9 binding sites, making these mRNAs candidates for direct transcriptional regulation by PAX9. Scanning for known PAX9 binding sequences [5’-SGTCACGCWTGANTGMA-3’, 5’-CGCGTGACCG-3’ [54], CD19-2(A-ins) 5’-GCGTGACCA-3’, and e5 5’-GCGGAACGG-3’] in the 1 kb upstream of the 1670 mRNAs using CentriMo analysis [55] reveals 3227, 2245, 2174, and 3885 potential PAX9 binding sites, respectively, in the 1 kb upstream of the 1670 mRNAs. This number of potential binding sites upstream of the 1670 mRNAs (~1.7 sites per gene) is similar to that observed in PAX9 ChIP experiments in the vertebral column of E12.5 mice (1.74 sites per gene, [54]). Additionally CentriMo enrichment analysis [55] of the sequence 1 kb upstream of each of the 1670 differentially expressed mRNAs reveals significant enrichment of 242 different DNA binding sequences, including the PAX3, PAX5, PAX6, and PAX7 DNA binding domains. As multiple PAX proteins can bind the same DNA sequence [56], this provides further evidence for PAX9 regulation of these 1670 mRNAs. These analyses therefore support the hypothesis that PAX9 regulates the levels of the mRNAs identified in our RNA-seq dataset.

In the RNA-seq dataset, many of the 1670 differentially expressed mRNAs have known roles in nucleolar function. For example, 156 (9.3%) have appeared in other genome-wide siRNA screens for nucleolar function (S1 Table, [34, 57, 58]). Additionally, 295 (17.7%) of the 1670 differentially expressed mRNAs code for proteins designated as nucleolar in at least 1 of 3 nucleolar databases (S1 Table, [50–52]). This is a significant enrichment in the expected number of nucleolar proteins, assuming that nucleolar proteins make up only 5% of the proteins in human cells [51]. Surprisingly, expression of most of the mRNAs encoding nucleolar proteins (184/295, 62.4%) was downregulated upon PAX9 depletion indicating that PAX9 is required to maintain normal levels of many mRNAs whose protein products are destined for function in the nucleolus (S1 Table).

To determine the mechanism of PAX9’s function in SSU biogenesis, we chose 5 candidates from the RNA-seq dataset to follow up on in greater detail. The mRNA levels for the 5 candidates were all downregulated after PAX9 depletion and all code for nucleolar proteins (Fig 2B and S1 Table, [50–52]). Four of the candidates (RPS6/eS6, RPS9/uS4, RPS28/eS28, and FBL) were chosen on the basis of literature suggesting a role for these proteins in SSU pre-rRNA processing in HeLa cells [59, 60]. Additionally, it was pertinent to analyze RPL5/uL18, as it has a known role in the p53-dependent nucleolar stress response [9]. qRT-PCR confirmed the RNA-seq results with reduced levels of each mRNA when PAX9 is depleted in MCF10A cells (Fig 2C). Interestingly, depletion of either RPS9/uS4 or RPS28/eS28 resulted in a decrease in nucleolar number from 2–3 to only 1 in our original siRNA screen [34]. Therefore, it is possible that the mechanism through which PAX9 depletion results in decreased nucleolar number relies upon reduced expression of RPS9/uS4 and/or RPS28/eS28 (Fig 2A).

We were able to confirm that depletion of 4 of the 5 tested candidates (RPS6/eS6, RPS9/uS4, RPS28/eS28, and FBL) in MCF10A cells resulted in pre-rRNA processing defects similar to that of PAX9 depletion (Fig 2D and 2E, S5 Fig). Only RPL5/uL18 did not give the 30S increase characteristic of PAX9 depletion, although this was expected given its known role in LSU pre-rRNA processing [10]. Additionally, depletion of several of the candidates individually resulted in significantly decreased global protein synthesis by the puromycin incorporation assay, similar to the effect seen after PAX9 depletion (Fig 2F and 2G). Puromycin incorporation was also reduced after RPS6/eS6 depletion, although it was not statistically significant (Fig 2F and 2G). Therefore, PAX9 may function as a transcription factor to directly or indirectly increase the expression of RPS6/eS6, RPS9/uS4, RPS28/eS28, and/or FBL (Fig 2A). As the proteins encoded by these mRNAs are required for SSU ribosome biogenesis (Fig 2), their depletion after PAX9 knockdown is a plausible mechanism through which PAX9 regulates pre-rRNA processing and global protein synthesis.

### RNAPII ChIP-seq analysis reveals decreased transcription of mRNAs encoding nucleolar proteins after PAX9 depletion

As the RNA-seq analysis confirmed that levels of 184 nucleolar mRNAs were decreased after PAX9 depletion (Fig 2), we sought to map how RNAPII distributes on genes using RNAPII ChIP-seq as a readout of transcription. Through this approach we aimed to untangle the effects of PAX9 depletion on RNAPII transcription from its effects on mRNA stability, since RNAPII ChIP-seq is able to detect genome-wide changes in RNAPII occupancy. As PAX proteins are able to both activate and repress target protein expression [15], we have included genes with both increased and decreased RNAPII occupancy in this analysis. Approximately 134 mRNAs were differentially occupied by RNAPII upon PAX9 knockdown compared to the non-targeting control siRNA (siNT) in MCF10A cells (fold change cutoff ≥ 2 or ≤ -2 and MaxTags ≥ 150) (S2 Table). Of these 134, 69 had decreased RNAPII occupancy, consistent with PAX9 acting as a transcriptional driver of these mRNAs.

**Fig 3:**
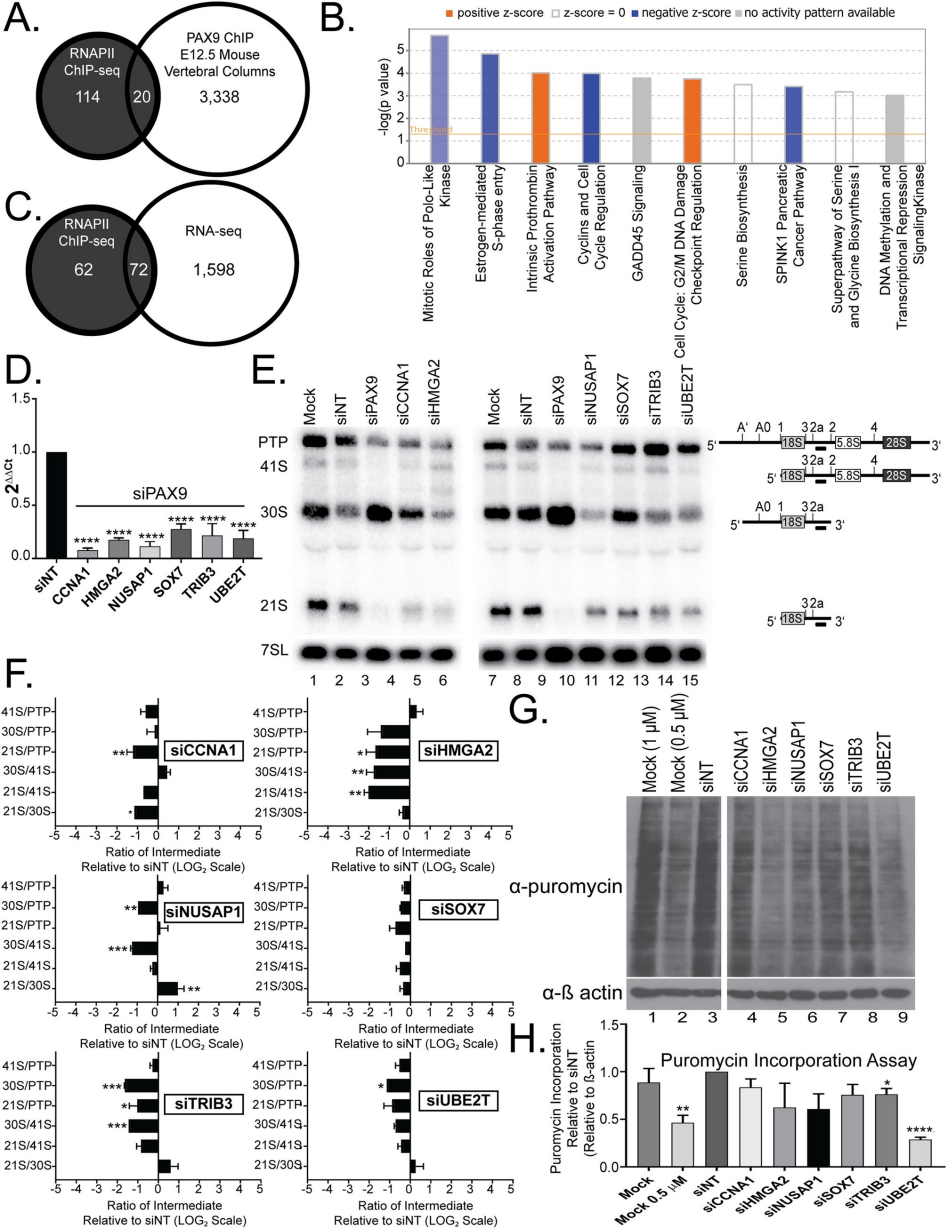
RNAPII ChIP-seq analysis reveals that PAX9 drives the transcription of mRNAs that code for nucleolar proteins. (A) Overlap of the genes differentially occupied by RNAPII when PAX9 is depleted in human MCF10A cells compared to the PAX9 ChIP-seq data from the vertebral columns of E12.5 mice [54]. (B) The 134 genes differentially occupied by RNAPII after PAX9 knockdown are enriched for cell cycle regulators. Comparative pathways analysis using Ingenuity Pathways Analysis (IPA) software (QIAGEN Inc., https://www.qiagenbioinformatics.com/products/ingenuitypathway-analysis) reveals both upregulated (orange) and downregulated (blue) pathways enriched in the list of 134 RNAPII ChIP-seq differentially occupied mRNAs. The comparative pathways analysis z score reflects the correlation between the observed expression of the mRNAs in each pathway and the predicted expression change based on existing literature. Pathways with a z score of 0 are shown as white and pathways with no activity pattern available are shown in gray. Only pathways enriched with a -log(p-value), which measures the enrichment of that pathway in the RNAPII ChIP-seq dataset, of ≥ 3 are shown. (C) Venn diagram showing that 72 genes overlap between the list of 134 genes that are differentially occupied by RNAPII after PAX9 knockdown in MCF10A cells and the list of 1670 mRNAs differentially expressed upon PAX9 knockdown as observed by RNA-seq in MCF10A cells. (D) qRT-PCR confirms depletion of the 6 candidates that exhibit both reduced occupancy in the RNAPII ChIP-seq dataset and reduced mRNA levels in the RNA-seq dataset after PAX9 siRNA knockdown in MCF10A cells (RNAPII ChIP-seq/RNA-seq candidate mRNAs). The levels of 6 mRNAs were quantified using qRT-PCR after depletion using siRNAs targeting either PAX9 or a non-targeting control siRNA (siNT). Data are shown as mean ± SEM. Three replicates using cells of different passage numbers, each with 3 technical replicates, were performed. Significance was calculated by One-way ANOVA using GraphPad Prism where **** p ≤ 0.0001, *** p ≤ 0.001, and ** p ≤ 0.01. (E) Northern blot analysis reveals small subunit (SSU) pre-rRNA processing defects after depletion of the 6 RNAPII ChIP-seq/RNA-seq candidate mRNAs in MCF10A cells. Representative northern blot after knockdown of the indicated siRNAs using probe P3. A probe for the 7SL RNA was used as a loading control. Pre-rRNA processing intermediates detected by probe P3 are shown to the right of the northern blot. PTP indicates the 47S, 45S, and 43S processing intermediates. (F) siRNA knockdown of 5 of the 6 tested RNAPII ChIP-seq/RNA-seq candidate mRNAs results in significant SSU pre-rRNA processing defects in MCF10A cells. Ratio analysis of multiple precursors (RAMP, [40]) quantitation of probe P3 northern blots in (E). Graph is mean ± SEM. N = 3. 2-way ANOVA. *** p ≤ 0.001, ** p ≤ 0.01, and * p ≤ 0.05. (G) Depletion of RNAPII ChIP-seq/RNA-seq candidate mRNAs results in reduced global protein synthesis. After 72 hours knockdown with the indicated siRNAs, MCF10A cells were pulsed with puromycin for 1 hour and protein was harvested. Western blotting with an anti-puromycin antibody and a β actin loading control was completed. Mock (no siRNA), mock at half the concentration of puromycin (0.5 μM) and siNT (non-targeting siRNA) are shown as negative controls. (H) Quantitation of 3 replicates using MCF10A cells of different passage numbers of the puromycin incorporation assay shown in (G) relative to siNT and the β actin loading control. Data are shown as mean ± SEM. N = 3. Significance was calculated by Student’s t-test using GraphPad Prism where **** p ≤ 0.0001 and ** p ≤ 0.01.

To assess the validity of the RNAPII ChIP-seq dataset, we again used CentriMo to identify potential PAX9 DNA-binding sites in the 1 kb upstream of the 134 genes with differential RNAPII occupancy [55]. Searching for the 5’ CGCGTGACCG 3’ PAX9 binding motif defined in [54] revealed 186 possible sites in the 1000 bp upstream of the 134 differentially occupied genes. Also, the known PAX9 DNA binding motifs CD19-2(A-ins) (5’-GCGTGACCA-3’) and e5 (5’-GCGGAACGG-3’) had 131 and 2 binding sites in these sequences, respectively. Additionally, Analysis of Motif Enrichment (AME)[61] identified the PAX5 and PAX6 DNA binding motifs as being significantly enriched in the 1 kb of sequence upstream of the 134 genes (p ≤ 0.05). Since multiple PAX proteins can bind the same motif [56], this suggests that this dataset does contain mRNAs that are regulated by PAX9. Of the 134 differentially occupied genes, 20 have also been shown to be differentially regulated by PAX9 directly in PAX9 ChIP-seq experiments on E12.5 WT vertebral column murine tissue (Fig 3A and S2 Table, [54]). These analyses confirm the ability of RNAPII ChIP-seq to detect changes in RNAPII-mediated transcription after PAX9 knockdown.

Based on the hypothesis that PAX9 acts as an RNAPII transcription factor for regulators of nucleolar function (Fig 2), we expected to detect changes in the RNAPII-mediated transcription of a number of mRNAs encoding nucleolar proteins after PAX9 depletion. Indeed, mRNAs coding for nucleolar proteins were enriched, with 46 of the 134 genes with differential RNAPII occupancy (34.3%) coding for nucleolar proteins in at least one of three databases (S2 Table, [50–52]). This is, again, higher than would be expected, assuming that nucleolar proteins account for approximately 5% of all cellular proteins [51]. Additionally, depletion of one gene with differential RNAPII occupancy (ANLN) resulted in decreased nucleolar number in our siRNA screen, similar to the phenotype seen after PAX9 depletion (Fig 1B) [34]. Notably, 18 of the 134 genes with differential RNAPII occupancy (13.4%) appeared in other genome-wide screens for ribosome biogenesis factors (S2 Table, [57, 58]). Another targeted screen investigated the effects of 2 of the 134 genes (TOP2A and CDCA8) on pre-rRNA processing when depleted by siRNA in HeLa cells [60]. However, depletion of neither gave the 30S pre-rRNA increase on northern blots characteristic of PAX9 depletion [60]. In all, the RNAPII ChIP-seq analysis confirmed that PAX9 drives the transcription of mRNAs encoding proteins critical for ribosome biogenesis.

In addition, both Gene Ontology (GO) enrichment [62–64] and Ingenuity Pathway Analysis [53] of the RNAPII ChIP-seq list show an enrichment of genes associated with cell cycle regulation (Fig 3B). This is consistent with PAX9 depletion resulting in a slight G1 accumulation as shown above (S3 Fig) and with alterations in ribosome biogenesis resulting in cell cycle inhibition [43, 44]. Therefore, the RNAPII ChIP-seq analysis supports the influence of PAX9 over the distribution of cells within the cell cycle as would be expected from a regulator of ribosome biogenesis [43–48].

Based on our RNA-seq results above (Fig 2 and S1 Table), we hypothesized that PAX9 may act as a transcription factor for RPS6/eS6, RPS9/uS4, RPS28/eS28, and/or FBL. Interestingly, although RNAPII occupancy for RPS6/eS6, RPS9/uS4, RPS28/eS28, and FBL was decreased to a slight extent in the RNAPII ChIP-seq dataset, the reduction was not statistically significant. However, it is possible that even such subtle changes in transcription levels for these essential proteins are sufficient, in combination, to cause the pre-rRNA processing inhibition seen upon PAX9 depletion. Additionally, the lack of significant changes in RNAPII occupancy for these mRNAs may support an indirect mechanism through which PAX9 regulates their levels, possibly by acting as an RNAPII transcription factor for proteins that affect the mRNAs’ stability (Fig 2A).

### Overlap of RNAPII ChIP-seq and RNA-seq differentially expressed genes

There was significant overlap between the RNA-seq and RNAPII ChIP-seq datasets. Over half (72/134) of the RNAPII differentially occupied mRNAs were also differentially expressed in the RNA-seq analysis (Fig 3C and Table 1). PAX9 may affect the transcription of these mRNAs either by directly binding to their promoters or by indirect methods such as affecting the transcription of another transcription factor (Fig 2A). Of these 72 overlapping genes, 39 genes had decreased mRNA expression in both analyses, consistent with PAX9 enhancing the levels of these mRNAs either directly or indirectly (Table 1). Of the 39 decreased and overlapping genes, 19 code for nucleolar proteins (Table 1, [50–52]). We have designated these 19 genes as the high-confidence list of potential PAX9 targets that affect ribosome biogenesis.

**Table 1:** List of mRNAs that are differentially expressed in both the RNAPII ChIP-seq and RNA-seq datasets, mRNAs with decreased expression in both lists, and mRNAs that have decreased expression in both lists and are also nucleolar in at least 1 database [50–52].

Overlap between RNA-seq and RNAPII ChIP-seq lists	Decreased expression in both lists	Decreased expression in both lists and Nucleolar
ALDH1A3	CCNA1	ALDH1L2
ALDH1L2	BIRC5	ARHGAP11A
AP5B1	MKI67	AURKB
ARHGAP11A	AURKB	CCNA1
ARHGAP30	CCNB1	CCNB1
ASPM	TOP2A	CDCA8
AURKB	MT2A	CDK1
BIRC5	STC2	DEPDC1
C15orf48	LOC100288637	HJURP
CCNA1	PIF1	HMGA2
CCNB1	HJURP	KIF18B
CD24	CEP55	MKI67
CDCA7	PHGDH	NUSAP1
CDCA8	ARHGAP11A	PCK2
CDH1	PECAM1	PHGDH
CDK1	NUSAP1	PTMS
CDKN1A	TRIB3	SOX7
CDKN3	NEIL3	TOP2A
CDSN	KIF18B	TRIB3
CENPM	CHAC1	
CEP55	PRC1	
CHAC1	CENPM	
CLDN4	PCK2	
COL1A1	CDKN3	
CPA4	PSAT1	
DEPDC1	NEK2	
DSC2	CDCA7	
EIF4EBP1	PLK4	
ELF3	SOX7	
ESPL1	ESPL1	
FBXO32	ASPM	
GPRC5A	CDK1	
HJURP	PTMS	
HMGA2	KCTD15	
HS3ST1	ALDH1L2	
KCNK15	EIF4EBP1	
KCTD15	CDCA8	
KIF18B	DEPDC1	
KLK10	HMGA2	
KLK7		
KRT16		
KRT80		
LMO7		
MACC1		
MAP2		
MKI67		
MT2A		
MUC16		
NEIL3		
NEK2		
NUSAP1		
OVOL1		
PCK2		
PECAM1		
PHGDH		
PIF1		
PLAUR		
PLK4		
PRC1		
PSAT1		
PTMS		
RAB11FIP1		
SCD5		
SERPINB2		
SLC2A12		
SOX7		
SPRR2D		
STC2		
SYTL2		
TOP2A		
TRIB3		
VSTM2L		

Further analysis of the mRNAs differentially regulated in both the RNA-seq and RNAPII ChIP-seq datasets validates PAX9 as a driver of these mRNA levels. GO analysis of the 72 genes present in both datasets again revealed enrichment of genes associated with cell cycle regulation as well as cell division [62–64]. As expected based on analysis of the whole RNAPII dataset above, CentriMo analysis of the 1 kb upstream of the 72 genes that appeared in both the RNAPII ChIP-seq and RNA-seq hit lists showed enriched DNA binding motifs for both PAX5 and PAX6 [55]. Therefore, these two unbiased approaches to detect changes in mRNA levels have revealed a high-confidence list of 19 nucleolar proteins for which PAX9 acts as a transcriptional driver.

From the high confidence list of 19 downregulated mRNAs that encode nucleolar proteins, we chose 5 (CCNA1, HMGA2, NUSAP1, SOX7, and TRIB3) to analyze in greater detail (Fig 3 and S6 Fig). Both CCNA1 and HMGA2 have been shown to be directly regulated by PAX9 in a ChIP-seq study of E12.5 murine intervertebral discs [54]. Interestingly, HMGA2 deletion in rabbits resulted in dwarfism (small stature being a common phenotype in diseases of impaired ribosome biogenesis) and altered craniofacial development [65]. We also chose to study UBE2T, as it is nucleolar and was shown to be downregulated in both datasets, although not to the same extent as the other chosen hits. UBE2T is essential for the Fanconi anemia DNA repair pathway which has been newly connected to ribosome biogenesis [66]. qRT-PCR confirmed depletion of all of these candidates when PAX9 is depleted in MCF10A cells (Fig 3D). We therefore hypothesized that depletion of one or multiple of these 6 mRNAs could provide the mechanism through which PAX9 regulates SSU biogenesis.

Interestingly, while individual depletion of none of the 6 tested candidates resulted in the same pre-rRNA processing defect as PAX9 depletion, most of the candidates did affect SSU pre-rRNA processing to some extent as revealed by northern blotting(Fig 3E and 3F and S6 Fig). Depletion of CCNA1 caused a similar processing defect as PAX9 depletion (the 21S pre-rRNA was significantly decreased relative to its 30S precursor and to the 7SL loading control), but the accumulation of the 30S pre-rRNA processing intermediate was not significant (Fig 3E and 3F and S6 Fig). Additionally, depletion of TRIB3 and UBE2T significantly decreased global protein synthesis, while the other candidates altered protein synthesis to a lesser, non-significant extent (Fig 3G and 3H). qRT-PCR confirmed depletion when each candidate is individually depleted for the northern blotting experiments (S7 Fig). It is therefore unlikely that depletion of any of these 6 proteins alone is causative of the specific pre-rRNA processing defects observed after PAX9 depletion. However, reduced levels of these mRNAs, in combination, may contribute to the overall impairment of protein synthesis that occurs when PAX9 is depleted in human cells.

### *pax9* depletion in *X*. *tropicalis* disrupts craniofacial development and neural crest patterning

PAX9 has an established role in craniofacial patterning in human populations [25]. Our discovery that PAX9 also has a role in ribosome biogenesis supports a link between these craniofacial phenotypes and ribosome biogenesis, as has been observed for other ribosomopathies [29, 67, 68]. In different models, ribosomopathies appear to have a predilection for the neural crest, a key component in facial development [14, 29, 69–75]. The neural crest is a population of cells that are induced at the neural plate border and migrate to an expansive area of the embryo including the face, and there develop into a remarkable array of different cell types. Multiple PAX proteins influence the development of neural crest-derived tissues [76]. Additionally, studies in both mice and stage 35 *X*. *laevis* embryos support a function for PAX9 in the developing neural crest because *pax9* expression during development is highest in the sclerotome and pharyngeal pouches [28, 77, 78]. In order to model PAX9 based craniofacial development and a role for the neural crest, we depleted *pax9* in *X*. *tropicalis* embryos, a well-established model for human ribosomopathies [70, 79, 80].

**Fig 4:**
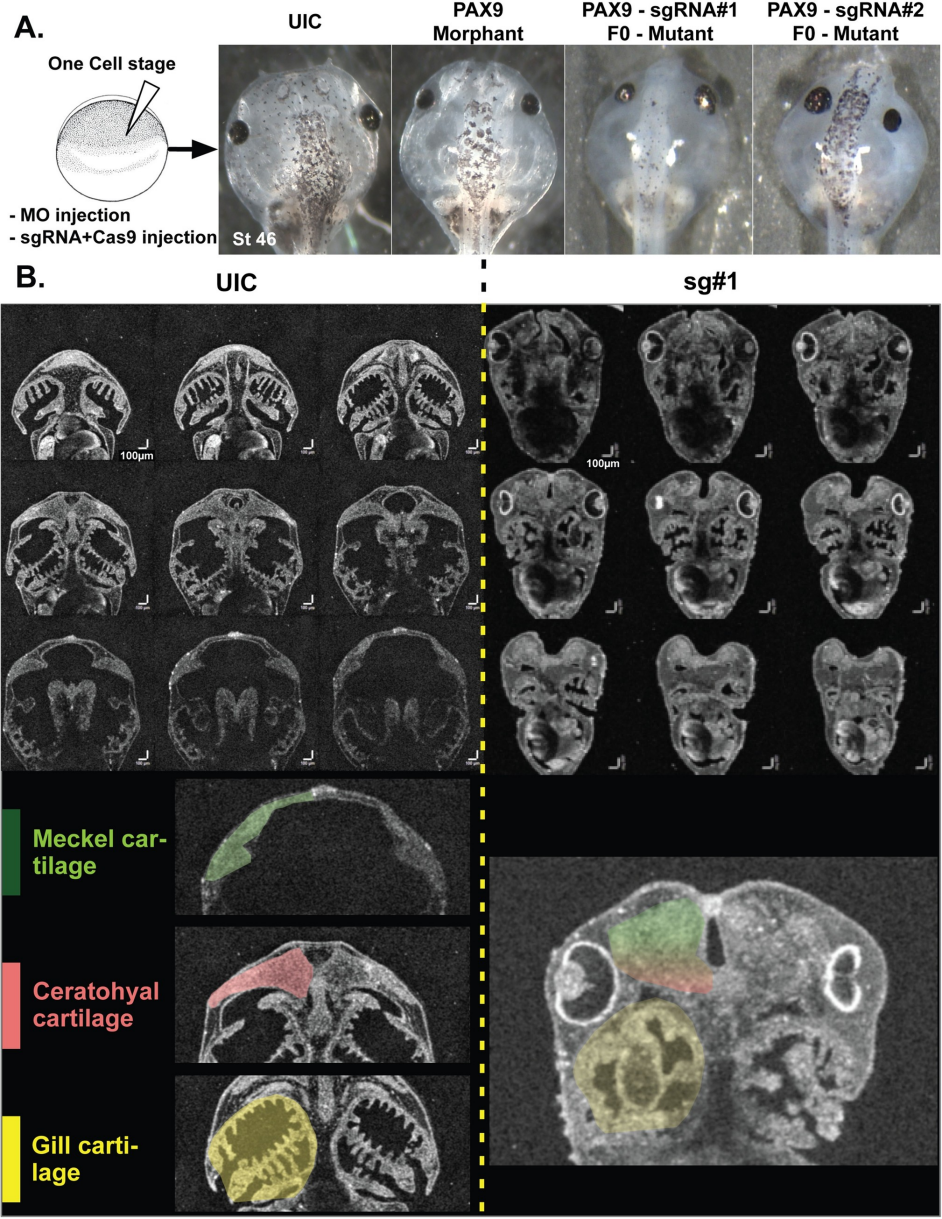
*pax9* depletion results in craniofacial dysmorphology in developing *X*. *tropicalis* embryos. (A) Representative craniofacial images of the indicated stage 45 embryos by stereomicroscopy. Pax9 knockdown with two non-overlapping CRISPRs (sg#1 and sg#2) or with a morpholino oligonucleotide (MO) demonstrates similar narrow, small facies. (B) F0 mutant embryos lacking Pax9 have malformed, dysplastic craniofacial cartilages. Craniofacial 3D imaging of the uninjected control (UIC, left) and F0 mutant sg#1 (right) by optical coherence tomography (OCT) imaging. The top panel shows montaged images along the dorsal ventral axis. The bottom panel shows the three major cartilages: Meckel’s (green), ceratohyal (red), and gill cartilages (yellow). Horizontal and vertical scale bars: 100μm.

We generated F0 embryos depleted of *pax9* by CRISPR-mediated gene modification or injected translation blocking morpholino oligonucleotides (MOs) into *X*. *tropicalis* embryos and analyzed their embryonic development compared to uninjected controls. Early embryonic development appeared normal. However, upon visual inspection, stage 45 (post fertilization day 4 @ 25°C) F0 mutant embryos generated using two different non-overlapping sgRNAs and morphant embryos exhibited facial abnormalities (Fig 4A and S8 Fig). On gross inspection, the distance between the eyes was shorter and more anteriorly displaced and the cartilage elements were smaller especially anteriorly such that the head was tapered, creating a “diamond” shape.

To examine the facial elements more closely, we used optical coherence tomography imaging (OCT). OCT is an imaging modality akin to ultrasonography but uses light rather than sound to generate an image. With OCT, we can readily detect the shape and size of the head structures [81]. There are three key cartilage elements that define the craniofacial structure of the embryos: Meckel’s, ceratohyal, and gill cartilages. Embryos depleted of *pax9* displayed profound size and shape abnormalities in these cartilages compared to uninjected controls (Fig 4B, compare CONTROL to sgRNA #1; S1 Movie). Normally, the Meckel’s and ceratohyal cartilages are broad and extend laterally widening the face of the embryo; however, in the case of *pax9* depleted embryos these cartilages were substantially smaller and abnormally shaped leading to narrow jaw structures.

### *pax9* depletion leads to pre-rRNA processing defects in *X*. *tropicalis* embryos

**Fig 5:**
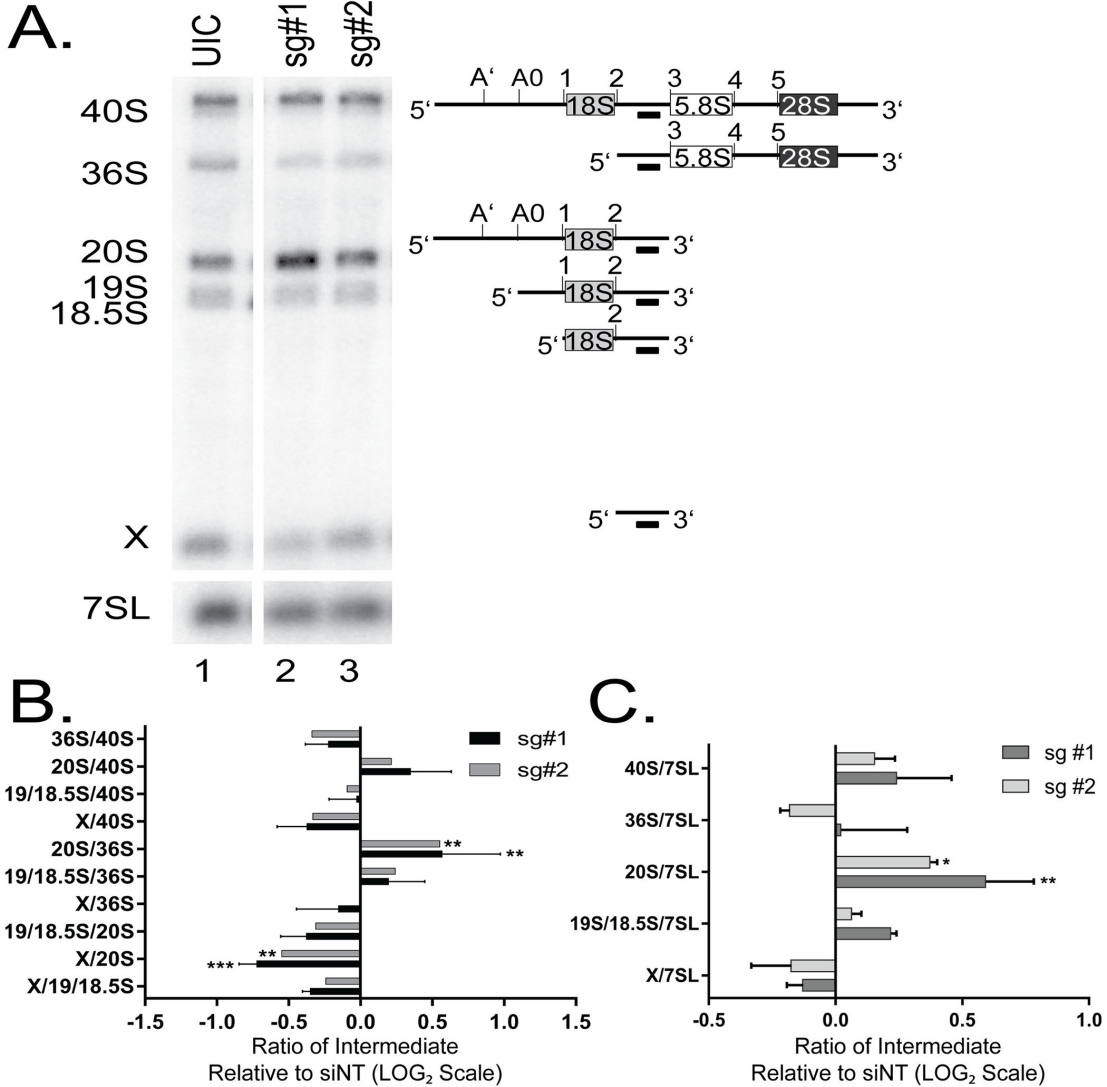
*pax9* depletion results in impaired ribosome biogenesis in *X*. *tropicalis*. (A) Northern blot hybridized with a probe for ITS1 (probe c from [70]). Processing intermediates visualized with this probe are shown to the right. Lane 1-uninjected control (UIC); lane 2–sg#1 embryos for F0 generation of Pax9 knockout; lane 3-sg#2 embryos for F0 generation of Pax9 knockout. RNA was harvested at stage 25. The X indicates the cleaved ITS1 RNA. (B) Ratio Analysis of Multiple Precursors (RAMP) [40] quantitation for the sg#1 (black) and sg#2 (gray) CRISPR knockouts of *pax9* shown in A. N = 3. Data are shown as the mean ± SEM, plotted on a LOG_2_ scale, relative to the UIC embryos. 2-way ANOVA. *** p ≤ 0.001 and ** p ≤ 0.01. (C) Quantitation for the sg#1 (black) and sg#2 (gray) CRISPR knockouts of *pax9* shown in A relative to a 7SL loading control. N = 3. Data are shown as the mean ± SEM, plotted on a LOG_2_ scale, relative to the UIC embryos. 2-way ANOVA. *** p ≤ 0.001 and ** p ≤ 0.01.

To test the extent to which ribosome biogenesis is affected by *pax9* depletion in *X*. *tropicalis*, we examined northern blots of RNA from embryos depleted of *pax9* in CRISPR F0 embryos (Fig 5). At stage 26, RNA was harvested from those embryos as well as from uninjected control embryos. Pre-rRNA processing was analyzed using a northern blot probe targeting ITS1 [70]. Knockout of *pax9* resulted in SSU pre-rRNA processing defects (Fig 5A; repetitions quantified in Fig 5B and 5C) similar to those seen in when the protein is knocked down in human tissue culture cells (Fig 1D). Therefore, PAX9’s role in human ribosome biogenesis is conserved from human tissue culture cells to the model organism, *X*. *tropicalis*.

### Pax9 is essential for Slug, Twist and Sox expression in the developing embryo

**Fig 6:**
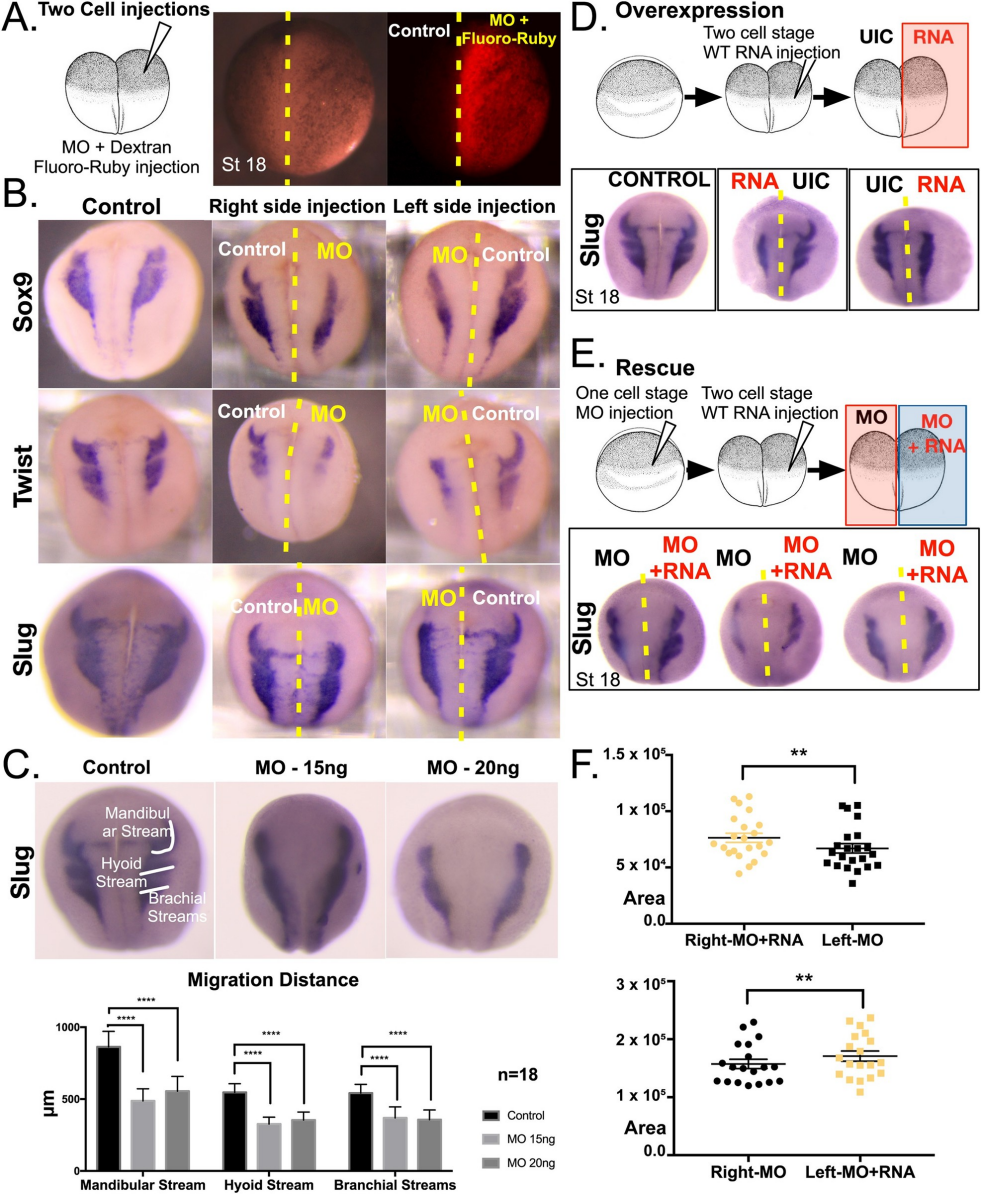
Neural crest cell marker gene expression is decreased in *pax9*-depleted *X*. *tropicalis* embryos. (A) Schematic depicting *in situ* hybridization experiments for neural crest markers. At the 2-cell stage, one cell was injected with a Pax9 MO and fluoro-ruby tracer dye. Embryos were grown to stage 18 before fixation and staining for Sox9, Twist, and Slug neural crest cell markers. (B) Reduced expression of Sox9, Twist, and Slug neural crest cell markers revealed by *in situ* hybridization in *Xenopus* embryos after Pax9 depletion. Control uninjected embryos are shown to the left. Embryos injected with the Pax9 MO and fluoro-ruby dye on the right side are shown in the middle. Embryos injected with the Pax9 MO and fluoro-ruby dye on the left side are shown to the right. (C) Reduced Slug expansion upon Pax9 depletion in *X*. *tropicalis* embryos. Ventral, Mid, and Dorsal Slug trajectories were quantified as indicated (μm, white lines) on both sides along the ventral dorsal axis in control uninjected embryos (left), embryos injected with 15 ng Pax9 MO at the one-cell stage (middle), and embryos injected with 20 ng Pax9 MO at the one-cell stage (right). N = 18. (D) Overexpression of human PAX9 mRNA alone in *X*. *tropicalis* embryos results in reduced Slug expression. At the 2-cell stage, human PAX9 mRNA was injected into 1 cell of the *X*. *tropicalis* embryos, with the other cell serving as an uninjected control (UIC). At stage 18, embryos were fixed and *in situ* hybridizations were performed to observe expression of the neural crest cell marker, Slug. (E) The injection of human PAX9 mRNA rescues Slug expression in Pax9-depleted *X*. *tropicalis* embryos, At the one-cell stage, embryos are injected with a MO targeting Pax9. At the 2-cell stage, one cell is injected with human PAX9 mRNA. Embryos were grown to stage 18 before *in situ* hybridizations were performed to monitor Slug expression. (F) Quantitation of Slug expression from rescue experiments shown in (E). Above: The side of the embryos that received both the PAX9 mRNA and MO (right side) had higher Slug expression than the side receiving only the MO (left side). Slug area manually segmented and measured with ImageJ. RNA injected side demonstrated increased expansion of slug. Below: The side of the embryos that received both the PAX9 mRNA and MO (left side) had higher Slug expression than the side receiving only the MO (right side). Slug area manually segmented and measured with ImageJ. RNA injected side demonstrated increased expansion of Slug. Student`s t-test; paired, used parametric test, ** p<0.01.

The neural crest is essential for the development of craniofacial structures. Neural crest cells are specified at the neural plate border before delaminating, migrating potentially large distances, and then differentiating into a plethora of cell types. Therefore, a defect in any of these steps can cause defects in craniofacial development. To examine how neural crest development is influenced by *pax9* depletion, we took advantage of the fate map of *X*. *tropicalis*, where embryos injected in one cell at the two cell stage can be selected such that either the right side or the left side is targeted. Therefore, the other side of the embryo acts as an internal control. We injected the MO targeting *pax9*, along with a lineage tracer (fluoro-ruby), in order to determine the affected and unaffected sides (Fig 6A). Embryos were raised to stage 18, sorted for the injected side, and then fixed. To examine neural crest cell fates, we performed whole mount *in situ* hybridization to detect neural crest marker gene expression for the mRNAs that encode the transcription factors Slug, Twist, and Sox9. In each case, the *pax9* depleted side had a reduction in neural crest marker gene expression (Fig 6B, S9 Fig). As we noted the reduction of Slug expression in PAX9 morphants, we then specifically examined each of the three neural crest streams (Mandibular, Hyoid and Brachial) and asked whether this is a global effect or specific to a particular neural crest stream. We injected varying doses of Pax9 MOs at the one cell stage and examined each stream using Slug expression. Depletion of Pax9 had a global effect on all three neural crest streams with significant reduction in Slug expansion (Fig 6C).

To demonstrate that Pax9 is necessary and sufficient to restore Slug expression and to further test the specificity of the MO targeting *pax9* in neural crest development, we sought to rescue Slug expression by co-injection of the human PAX9 mRNA. Because overexpression of the human PAX9 mRNA alone led to a reduced Slug signal, it was necessary to titrate the mRNA dose to a level where it caused no visible defects on embryogenesis (5 pg per embryo, Fig 6D, S10 Fig). To test for mRNA rescue, we injected the Pax9 MO at the one cell stage followed by human PAX9 mRNA injection (with a fluoro-ruby tracer) into one cell at the two cell stage (Fig 6E). At this mRNA dose, we observed an increase in Slug signal on the RNA injected side compared to the side that only received the MO, further verifying the specificity of our knockdown strategy (Fig 6E).

### *pax9* F0 mutants display increased apoptosis

**Fig 7:**
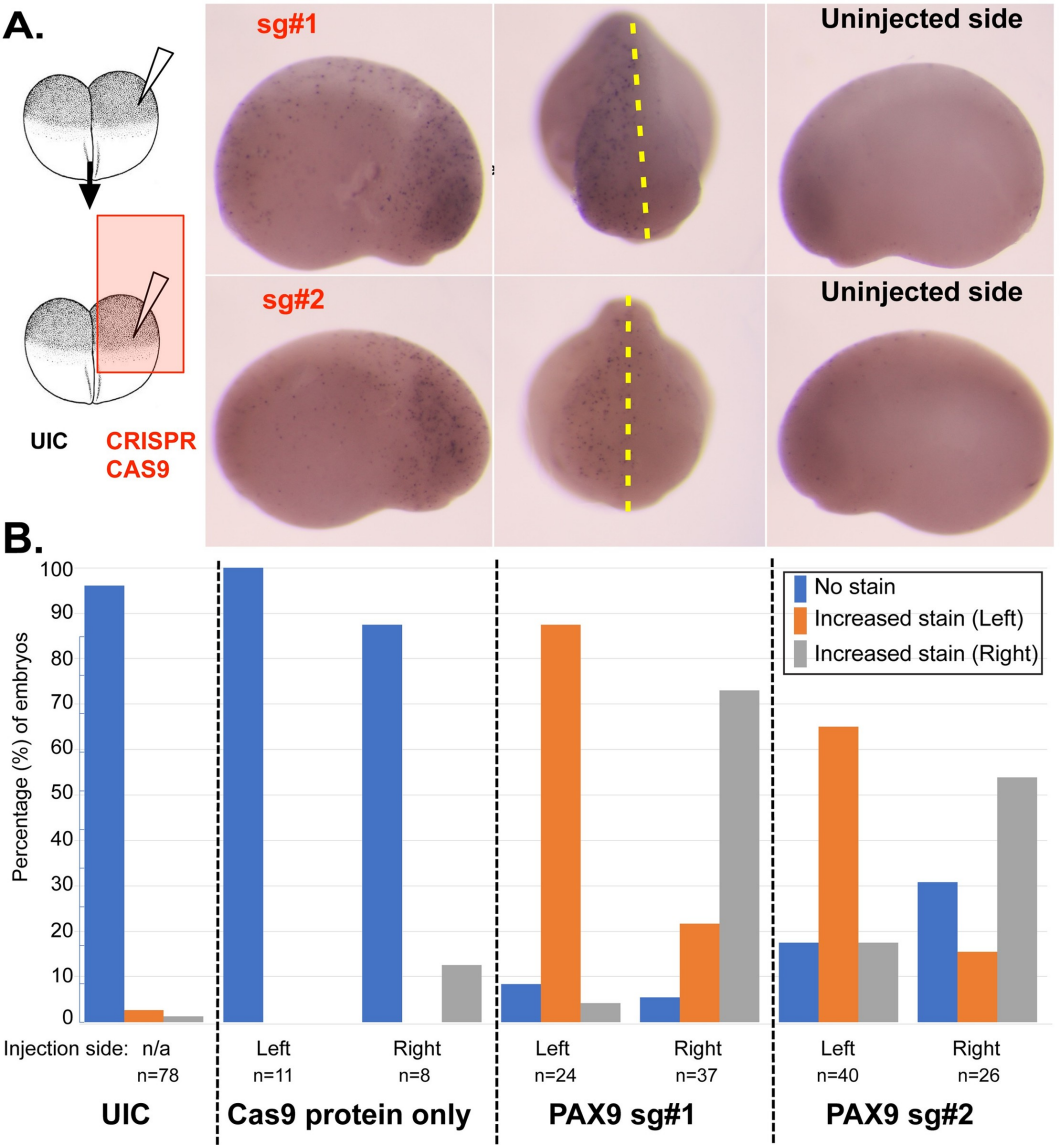
*pax9* depletion in *X*. *tropicalis* leads to increased apoptosis. (A) *pax9* depletion causes increased TUNEL staining in the developing embryo. At the two-cell stage, one side of the *X*. *tropicalis* embryos were injected with one of two non-overlapping CRISPRs sgRNAs (#1 and #2) targeting *pax9* and with CAS9 protein. TUNEL staining was performed at stage 20. For both CRISPR sgRNAs, increased TUNEL staining was observed on the half of the embryo that was depleted of Pax9 (shown to the left) as compared to the uninjected control half of the embryo (UIC, shown to the right). (B) Quantitation of the TUNEL staining shown in (A). Embryos were injected at the 2 cell stage as indicated below the graph: either uninjected control (UIC; negative control), Cas9 protein alone (negative control), PAX9 CRISPR sgRNA #1 (250 pg), or PAX9 CRISPR sgRNA #2 (250 pg). In all cases, 1 side of the embryo remained an uninjected control. Data were analyzed by comparing the amount of TUNEL staining on the injected side of the embryo to the amount of TUNEL staining on the uninjected side of the embryo. The percent of embryos with increased TUNEL staining on either the left side (orange), right side (gray), or without increased staining (“normal”, blue) were quantified for each injection condition (key shown to the right of the figure).

Since our results in MCF10A cells suggest that PAX9 is required for human ribosome biogenesis, we asked whether this loss of Slug expression reflects increased cellular apoptosis. In other ribosomopathies, apoptosis in neural crest cells leads to craniofacial malformations [8, 70, 71, 73, 75]. Therefore, we again performed knockout of *pax9* on either the right or left side of the embryo using the CRISPR/Cas9 system, leaving the other side of the embryo to serve as an uninjected control. We then fixed the embryos at st18-22 and performed TUNEL staining to detect apoptosis. Negative control embryos (UIC and embryos injected with Cas9 protein alone) reveal no differences in TUNEL signal (Fig 7A-B). Interestingly, however, embryos injected with either CRISPR sgRNA (#1 or #2) targeting Pax9 had increased TUNEL staining that was specific to the side of the embryo that had received the sgRNA (Fig 7A and 7B). Analysis of p53 levels by western blot revealed that p53 is stabilized in embryos depleted of Pax9 and in human RKO cells, lending insight into the potential mechanism of apoptosis (S11 Fig). This suggests that the craniofacial defects observed upon *pax9* depletion are mediated by increased apoptosis, similar to what has been seen when other proteins required for ribosome biogenesis are depleted in the developing embryo [8, 29, 70, 71, 73, 75].

## Discussion

PAX9 regulates human ribosome biogenesis by acting as an RNAPII transcription factor to influence the expression of multiple mRNAs required for pre-rRNA processing and global protein synthesis (Fig 2A). Depletion of PAX9 disrupts nucleolar structure so that the number of nucleoli is reduced from 2–3 to only 1 (Fig 1B). Additionally, PAX9 depletion results in SSU pre-rRNA processing defects and reduced global protein synthesis (Fig 1D–1F). As measured by RNA-seq, PAX9 depletion results in changes in the levels of a number of mRNAs (Figs 2and 3, S1 Table). Several of these differentially expressed mRNAs play roles in pre-rRNA processing and global protein synthesis (Fig 2). Interestingly, Pax9’s role in ribosome biogenesis is conserved to *X*. *tropicalis* (Fig 5). *X*. *tropicalis* embryos depleted of Pax9 have craniofacial malformations and increased cellular apoptosis (Figs 4 and 7). This is a consistent finding in other ribosomopathies that have been modeled in vertebrates and further highlights how depletion of ribosome biogenesis factors during development can cause congenital malformation. We have found a critical new role for the RNAPII transcription factor, PAX9, that connects the production of ribosomes to development of the face.

Thus far, our attempts to perform PAX9 ChIP-seq in human MCF10A cells have been unsuccessful due to a lack of ChIP-quality antibodies. Regardless, analysis of existing PAX9 ChIP-seq data from E12.5 WT vertebral column murine tissue does support a direct binding role for PAX9 for 265 of the 1670 differentially expressed genes (S1 Table, [54]). Of the 11 chosen candidates analyzed here, RPL5, CCNA1, and HMGA2 represent potential direct PAX9 binding targets based on the data from embryonic mouse vertebral columns [54]. Interestingly, individual depletion of none of these potentially direct targets revealed the same processing defect as PAX9 depletion (Fig 3E and 3F). It is therefore unresolved whether PAX9 acts indirectly or directly to regulate the levels of the other 8 candidate SSU biogenesis factors, some of which do contribute to the pre-rRNA processing defects. It is possible that these are direct targets of PAX9 that were not identified in the ChIP-seq dataset due to differences in model organism, tissue, and/or because the RNAPII ChIP-seq method was not saturating. It is likely that multiple factors contribute to both the stability and transcription of nucleolar mRNAs after PAX9 depletion. Therefore, future studies will be needed to tease apart the precise signaling mechanism through which PAX9 affects depletion of these ribosome biogenesis factors.

Several mRNAs, including the 5 candidates further examined in Fig 2, appeared in only the RNA-seq list. The transcription of these mRNAs may yet be regulated by PAX9, as neither genome-wide approach is saturated. Additionally, the stability of their mRNAs may be affected by PAX9 depletion. Crossing the RNAPII ChIP-seq dataset with 4 RNA binding protein databases [82–85] revealed 4 potential RNA binding proteins (NEIL3, MKI67, HIST1H1B, and NUSAP1). NUSAP1 was further examined here (Fig 3). However, depletion of NUSAP1 did not result in the same pre-rRNA processing defect as PAX9 depletion, suggesting that this is not the singular factor controlling mRNA stability. Additionally, depletion of MKI67 has previously been reported to have no effect on pre-rRNA processing [86]. Therefore, the mechanism(s) regulating the potential instability of these mRNAs remain undefined.

We have also confirmed the previously observed role of Wnt signaling in PAX9 function with these datasets (S4 Fig). Dickkopf WNT Signaling Pathway Inhibitor 1 (DKK1), a negative regulator of Wnt signaling, shows increased expression after PAX9 depletion in the RNA-seq dataset. Interestingly, increased expression of DKK1 was also found in a recent RNA-seq analysis of palatal shelves of E13.5 *Pax9*^*-/-*^ mice [30]. PAX9 binds to a region close to the DKK1 transcription start site to repress its transcription [33]. Inhibition of Dkk1 rescues the cleft palates of *Pax9*^*-/-*^ mice [30, 33]. One additional study has also suggested a role for the related EDAR/NF-κB signaling pathway in cleft palate formation in *Pax9*^*-/-*^ mice [32]. However, this pathway was not enriched in any of our datasets by Ingenuity Pathways Analysis (IPA, [32]). Interestingly, treatment of *Pax9*^*-/-*^ mice with Wnt inhibitors or a monoclonal antibody against EDAR did not rescue a number of phenotypes, including arrested tooth development, limb defects, and lethality just after birth [30–33]. This suggests that pathways other than Wnt signaling are contributing to the defects seen in *Pax9*^*-/-*^ mice, leaving open the possibility that ribosome biogenesis defects contribute to these other developmental anomalies.

Given the ubiquitous requirement for ribosomes in all growing cells, the tissue specific defects of ribosomopathies remain intriguing. Importantly, depletion of PAX9 in both *X*. *tropicalis* embryos and 3 human tissue culture cell lines leads to SSU pre-rRNA processing defects, strongly supporting the idea that PAX9 is involved in ribosome biogenesis in many different cell types. Once again, the developing face appears to be particularly sensitive to disruptions in ribosome biogenesis. While ribosome biogenesis is ubiquitous, *pax9* expression varies during embryonic development. At key stages (st 14–18), both RNA-seq and RT-PCR data demonstrate that *pax9* transcripts in whole embryos are low in abundance, while at later stages *pax9* is expressed at the margin of the branchial arch and the pharyngeal endoderm [77, 87]. Therefore, whether *pax9* is specifically expressed in the neural crest or acts non-autonomously in tissues adjacent to the neural crest is uncertain. Future lineage tracing experiments may determine if *pax9* functions autonomously or non-autonomously in developing neural crest cells. Regardless, increased apoptosis at an early window in embryonic development in the frog by depletion or genetic knockout of *pax9* leads to malformation of the major cartilages of the face.

Many instances of *PAX9* mutation in humans are autosomal dominant, where one allele is often functionally null, similar to what is seen in other known ribosomopathies. Indeed, many ribosomopathies also affect craniofacial and cardiac development (reviewed in [29]). However, *PAX9* mutations often result in oligodontia, which affects tooth development but does not usually affect the rest of the craniofacial skeleton. In contrast, in *X*. *tropicalis* we find severe craniofacial malformations after *pax9* depletion, perhaps due to the method of knockdown in *X*. *tropicalis*. One possibility is that further analyses of human populations world-wide may uncover additional homozygous mutations in PAX9 that result in more severe craniofacial defects akin to those observed in our *X*. *tropicalis* model system. Alternatively, the craniofacial and/or cardiac defects could occur as a result of other functions of PAX9. Taken together, these results suggest that the novel role for PAX9 in ribosome biogenesis that we have found here may contribute to the tooth and craniofacial malformations seen in humans with *PAX9* mutations, an unexpected finding given the other functions of many of the members of the PAX gene family.

## Materials and Methods

### Ethics statement

*X*. *tropicalis* were maintained and cared for in our aquatics facility, in accordance with Yale University Institutional Animal Care and Use Committee protocols.

### Cell lines and media

MCF10A cells (ATCC CRL-10317) were grown in Dulbecco’s modified Eagle’s medium/Nutrient mixture F-12 (DMEM-F12, Gibco 1130–032) supplemented with 5% horse serum (Gibco 16050), 20 ng/mL epidermal growth factor (Sigma E4127), 0.5 μg/mL hydrocortisone (Sigma H0135), 100 ng/mL Cholera toxin (Sigma C8052), and 10 μg/mL insulin (Sigma I1882). HEK293FT (a generous gift from P. Glazer) and RKO (ATCC CRL-2577) cells were grown in DMEM (Gibco 41965–062) with 10% FBS. All cell lines were grown at 37°C in a humidified incubator with 5% CO_2_.

For all experiments, except for the original siRNA screen and luciferase assay, siRNA knockdown was performed in 6-well plates with 1x10^5^ cells per well. After 24 hours, siRNAs (30 nM final) were reverse transfected for 72 hours using Lipofectamine RNAimax as per the manufacturer’s instructions. The original siRNA screen methods can be found in [34]. The luciferase assay was performed as in [34]. All siRNAs were purchased from Dharmacon (siGENOME, S2 Table).

### Luciferase assay

The dual-luciferase reporter assay was performed as in [34] using pHrD-IRES-Luc plasmid from [39] and *Renilla* control plasmid from [38]. Four replicates of MCF10A cells of different passage numbers were performed. Statistical significance was calculated by Student’s t test using GraphPad Prism, version 7.01 for Windows, GraphPad Software, La Jolla California USA, www.graphpad.com. **** p ≤ 0.0001.

### Northern blots

After 72 hours of siRNA knockdown, RNA was harvested using TRIzol (Life Technologies 5596018) as per the manufacturer’s instructions. Further details and probe sequences are as published in [34]. Statistical significance (p < 0.05) was calculated by 2-way ANOVA with Sidak’s multiple comparisons test using GraphPad Prism, version 7.01 for Windows, GraphPad Software, La Jolla California USA, www.graphpad.com. **** p ≤ 0.0001, *** p ≤ 0.001, ** p ≤ 0.01, and * p ≤ 0.05. Ratio analysis of multiple precursors (RAMP) was performed as previously described [40]. At least three replicates using cells of different passage numbers were performed for each northern blot.

### Puromycin incorporation assay

The puromycin incorporation assay was performed as in [34]. Three replicates using MCF10A cells of different passage numbers were performed. Significance was calculated by One-way ANOVA using GraphPad Prism, version 7.01 for Windows, GraphPad Software, La Jolla California USA, www.graphpad.com. **** p ≤ 0.0001 and *** p ≤ 0.001.

### Polysome Profiling

Polysome profiles were performed after 72 hours of knockdown using siRNAs as described above. Cells were washed 3 times in 100 μg/mL cycloheximide in PBS. Cells were then lysed in polysome lysis buffer (15 mM Tris, pH 7.5, 5 mM MgCl_2_, 150 mM NaCl, 2 mM DTT, 100 μg/mL cycloheximide, 1% Triton-X 100, Roche protease inhibitor cocktail). Samples were spun at max speed to remove insoluble material, and protein content normalized. Two hundred μg total protein was loaded onto 5–50% sucrose gradients (15 mM Tris, pH 7.5, 5 mM MgCl_2_, 150 mM NaCl, 2 mM DTT, 100 μg/mL cycloheximide, 5 or 50% RNase-free sucrose). Gradients were centrifuged in a SW-41Ti rotor for 90 min at 36,000 rpm at 4°C and sampled using a Biocomp Gradient Station with constant monitoring of optical density (OD) at 254 nm.

### Cell cycle analysis

After 72 hours of siRNA knockdown as described above, MCF10A cells were pelleted at 400 x g for 3 minutes at 4°C. Samples were washed with PBS-B [PBS with 1% BSA (Sigma, A9647)] before fixation in 4% formaldehyde (J.T. Baker 2106–01) in PBS for 20 minutes at room temperature. After washing with PBS-B and centrifugation at 400 x g for 3 minutes, cells were resuspended in 90% ice-cold methanol and incubated on ice for 30 minutes. After washing twice with PBS-B, cells were resuspended in RNase/PI (BD Pharmgen, Catalog No. 550825) and incubated for 15 minutes at room temperature. Flow data was collected at the Yale Flow Cytometry Facility on a BD LSRII Flow Cytometer and analyzed using FlowJo CE. The experiment was performed 3 times using cells of different passage numbers.

### qRT-PCR

After 72 hours of siRNA knockdown, RNA was extracted using TRIzol (Life Technologies 5596018) as per the manufacturer’s instructions. cDNA synthesis was performed using the iScript gDNA Clear cDNA Synthesis Kit from Bio-Rad (Cat. No. 172–5035), and the SYBR Green reagent was also purchased from Bio-Rad (Cat. No. 1725121). All A_260/230_ values were above 1.7 prior to cDNA synthesis. Melt curves were performed for each sample to verify the amplification of a single product. Cycling parameters using the Applied Biosystems StepOne Plus are as follows: initial denaturation at 95°C for 30s and 40 cycles of 95°C for 15s and 55°C or 58°C for 30s. Melt curve analysis: 95°C for 15s, then 55°C or 58°C for 1 min, and a gradual increase in temperature (0.3°/15s) to 95°C. Analysis was completed using the comparative C_T_ method (ΔΔCT). Three replicates using cells of different passage numbers were performed, each with three technical replicates were measured for each experiment. Significance was calculated by One-way ANOVA with Dunnett’s multiple comparisons test or Student’s t test, as indicated in the figure legends, using GraphPad Prism, version 7.01 for Windows, GraphPad Software, La Jolla California USA, www.graphpad.com. **** p ≤ 0.0001, *** p ≤ 0.001, ** p ≤ 0.01, and * p ≤ 0.05. Primer sequences are shown in S3 Table. Whenever possible, published primers and intron-spanning primers were used.

### Western blotting

In human tissue culture cells, after 72 hours of siRNA knockdown, protein was harvested by scraping, rinsed in PBS, and lysed by vortexing in AZ lysis buffer (50 mM Tris pH 7.5, 250 mM NaCl, 1% Igepal, 0.1% SDS, 5 mM EDTA pH 8.0) containing protease inhibitors (cOmplete Protease Inhibitor Cocktail, Roche, 11697498001) for 15 minutes at 4°C. The lysate was spun at 21,000 x g for 15 minutes at 4°C. The supernatant was removed, and total protein was quantified by Bradford assay (Bio-Rad). Samples were analyzed via SDS-PAGE. Antibodies used include α-p53 (1:5,000 Santa Cruz, sc-126 HRP), α-β-actin (1: 30,000 Sigma-Aldrich A1978), α-PAX9 (1:1,000, Cell Signaling Technology D9F1N), α-vinculin (1:20,000 Abcam ab18058), and α-puromycin (1:10,000 Kerafast 3RH11). HRP conjugated secondary antibodies include α-mouse (1:10,000 GE Healthcare NXA931) and α-rat (1:10,000 GE Healthcare NA935V). Images acquired either by film developing or by digital imaging using the BioRad ChemiDoc Imaging System.

For *X*. *tropicalis* western blotting, protein was harvested according to the TRIzol (Life Technologies 5596018) protocol for 2 replicates, and as in [70] for 1 replicate. Samples were analyzed via SDS-PAGE. Antibodies included α-p53 (1:800, Thermo Fisher MA1-12549) and α-GAPDH (1:5,000 Ambion AM4300). HRP conjugated secondary antibodies include α-mouse (1:10,000 GE Healthcare NXA931).

### RNA-seq

After 72 hours of siRNA knockdown in MCF10A cells using either siRNAs targeting PAX9 or a non-targeting control (siNT), RNA was harvested using TRIzol according to the manufacturer’s instructions. Agilent BioAnalyzer analysis revealed high quality RNA, with RIN numbers of 10, 10, and 10 for siNT samples and 10, 9.8, and 9.9 for siPAX9 samples. The Yale Center for Genomic Analysis synthesized the PolyA+ mRNA library following Illumina mRNA sample preparation guidelines and also performed the single-end RNA sequencing using the Illumina HiSeq 2500 sequencing system.

Total reads for each of the 6 samples ranged from approximately 19.8 million to 35.2 million, and the average read quality ranged from 36.28/40 to 36.43/40 with a read length of 76 nt. Data analysis was performed using Partek Flow software (version 7.0 Copyright; 2018, Partek, Inc., St. Louis, MO, USA). Reads were aligned to the genome (UCSC hg19) using Bowtie 2 [88] and quantified to the transcriptome using Cufflinks [89]. mRNAs were considered differentially expressed if the fold change was ≥ 2 or ≤ -2 and the q value ≤ 0.05. The functional analyses were generated through the use of IPA (QIAGEN Inc., https://www.qiagenbioinformatics.com/products/ingenuity-pathway-analysis, [53]).

All RNA-seq files are available from the Gene Expression Omnibus (GEO) and are accessible at the GEO Series accession number GSE154764 (https://www.ncbi.nlm.nih.gov/geo/query/acc.cgi?acc=GSE154764).

### RNAPII ChIP-seq

After 72 hours of siRNA knockdown of PAX9 or a non-targeting control siRNA (siNT), MCF10A cells were fixed in 10% fixing solution (11% formaldehyde, 0.1 M NaCl, 1 mM EDTA (pH 8.0), and 50 mM HEPES (pH 7.9)) for 15 minutes at room temperature. Cross-linking was stopped with 5% v/v 2.5 M glycine at room temperature for 5 minutes. Cells were washed once in PBS-Igepal (0.5%). Cells were washed again in PBS-Igepal (0.5%) with 1 mM PMSF. The cells were centrifuged at 800 x g, the supernatant removed, and the cell pellet was flash frozen on dry ice. Further steps of the TranscriptionPath RNAPII ChIP-seq protocol were performed by Active Motif. Chromatin immunoprecipitation was completed using an RNAPII antibody (Active Motif, Clone H4B, Cat. No. 39097). Sequencing was performed using the Illumina NextSeq 500 sequencing system with 75 nt sequence reads.

Data analysis was completed by Active Motif. The total number of reads per sample ranged from 35 to 38 million. The total number of reads aligned to the hg38 reference genome, after removal of duplicates, ranged from approximately 14 million to 18 million. Tags were normalized by random sampling to the sample with the smallest number of unique alignments without duplicate reads (14,165,891). Peak calling was performed using SICER 1.1 with a FDR of 1x10^-10^ and a gap size of 600 base pairs. The number of intervals per sample was approximately 24 million. mRNAs were considered differentially expressed if the fold change was ≥ 2 or ≤ -2, and the max tags (5’ ends of aligned reads) value was ≥ 150. The functional analyses were generated through the use of IPA (QIAGEN Inc., https://www.qiagenbioinformatics.com/products/ingenuity-pathway-analysis) (40).

All RNAPII ChIP-seq files are available from the Gene Expression Omnibus (GEO) and are accessible at the GEO Series accession number GSE154764 (https://www.ncbi.nlm.nih.gov/geo/query/acc.cgi?acc=GSE154764).

### X. tropicalis

*X*. *tropicalis* were maintained and cared for in our aquatics facility, in accordance with Yale University Institutional Animal Care and Use Committee protocols (2015–11035). Embryos were produced and raised to developmental stages described in Nieuwkoop and Faber 1994 according to established protocols [90]. Experiments are performed at Stage 18 (19 hr. 45 min pf @ 23°C), Stage 25 (1 day, 3 hr. 30 min pf @23C), Stage 40 (2 days, 18 hr. pf @23C) and Stage 46 (4 days, 10 hr. pf @ 23°C). Experiments in which the total death rate was higher than 15% were rejected in both MO or CRISPR knockdowns.

### MO knockdown in *X*. *tropicalis*

Following *in vitro* fertilization and as described previously [91], we injected 15–20 ng MO targeting the Pax9 AUG start site (Gene Tools, LLC). The sequence of the MO is as follows: 5’- TTCCCCGAAAGCGGGTTCCATTGCT -3’. Following injections, embryos were raised at 23˚C to stage 17–18 for *in situ* hybridization and stage 46 for craniofacial assessment with OCT imaging.

### CRISPR-CAS9 knockdown with RNA rescue in *X*. *tropicalis*

CRISPR/Cas9-mediated genome editing in *X*. *tropicalis* F0 embryos was carried out as previously described [92]. Two non-overlapping CRISPRs sgRNA were designed based on the v9.1/xenTro9 gene model on Xenbase and UCSC Genome Browser:

- Pax9 CRISPR sgRNA #1 oligo sequence targeting Exon2/12: taatacgactcactataGGGTGATGGAGCGAATCCCAgttttagagctagaa
- Pax9 CRISPR sgRNA #2 oligo sequence targeting Exon3/12: taatacgactcactataGGGCGAGGTGTCACTGACTAgttttagagctagaa

Following *in vitro* fertilization, we injected 250pg of sgRNA with 1.6 ng of Cas9 protein (CP03, PNA Bio) at the 1-cell stage. Alternatively, we injected 125 pg of sgRNA with 0.8 ng Cas9 protein into one cell at the 2-cell stage using standard methods [90, 93]. Following injections, embryos were raised at 23˚C to stage 17–18 for *in situ* hybridization, stage 25 and 40 for northern blotting, and stage 46 for craniofacial assessment with OCT imaging. Tetramethylrhodamine Dextran (D1817—Thermofisher) were co-injected with CRISPR for lineage tracing. We confirmed left or right sided injections using a Zeiss Lumar fluorescence stereomicroscope.

To genotype F0 CRISPR embryos, we amplified the CRISPR target sites using the following PCR primers:

Primer_sgRNA#1_Forward TGGCCCAACAGAACCACTAT
Primer_sgRNA#1_Reverse TCTATCTGTGTGCCGACTTG
Primer_sgRNA#2_Forward TGATAGAATAGTGCAATGGAAACA
Primer_sgRNA#2_Reverse CCACAGCGCTGCTAATAGACT

Amplicons were Sanger sequenced and analyzed using ICE (https://ice.synthego.com/#/) to identify the frequency and type of indels.

### Image acquisition and optical coherence tomography (OCT) imaging

A polystyrene petri dish (35 X 10mm) bottom was coated with wax based non-hardening clay (Van Aken Claytoon) and a well (0.5cm diameter) was formed at the center. Stage 46 embryos were immobilized in 1/9 x MR solution with benzocaine and positioned in the center of the well with their ventral side facing the OCT beam. Embryos were imaged using a Thorlabs Ganymede SD-OCT System 900 nm Center Wavelength. Three dimensional images of the *X*. *tropicalis* craniofacial structures were obtained. Cartilage length and area was measured as previously described [81]. We used ThorImage OCT v5.0.1 (Thorlabs GmbH, Luebeck) and FIJI (Image J) software for measurements [94]. Stereomicroscopy images were obtained using a Nikon SMZ745T stereomicroscope fitted to an Excelis Accu-Scope camera.

### Whole mount *in situ* hybridization (ISH)

Digoxigenin-labeled antisense probes for *twist* (IMAGE:7025399), *sox9* (TNeu111f21) and *slug* (TNeu008a21) mRNAs were transcribed with the T7 High Yield RNA Synthesis Kit (E2040S, New England Biolabs). Embryos were fixed in MEMFA (100 mM MOPS (pH 7.4), 2 mM EGTA, 1 mM MgSO_4_, 3.7% (v/v) formaldehyde) for 2 hrs. at room temperature and sequentially dehydrated into 100% Methanol. ISH was performed per previously described protocol [91].

These experiments were repeated at least three times on different days. In Fig 6C for the quantification of Neural Crest Development, we used FIJI (Image J) software for measurements [94]. Stream width in experiments was measured at three different positions and then averaged for each embryo. For significance, we compared the width of each stream using unpaired, non-parametric, Mann-Whitney U test (GraphPad Prism, version 8 for MacOS, GraphPad Software, La Jolla California USA, www.graphpad.com), **** p ≤ 0.0001, *** p ≤ 0.001, ** p ≤ 0.01, and * p ≤ 0.05.

### TUNEL staining

Terminal deoxynucleotidyl transferase dUTP nick end labeling (TUNEL) staining detects the DNA breaks formed during apoptosis. We used a Terminal Deoxynucleotidyl Transferase (TdT) (Invitrogen Cat #: 10533-0651X TdT Buffer) kit and Digoxygenin-dUTP (Roche Cat #: 11-093-088-910), following a previously published protocol [95].

### Northern blots on *X*. *tropicalis* RNA

Northern blotting was performed as described above for mammalian cells, with the following changes. At stage 25, RNA was harvested from injected embryos. Embryos were dissolved in TRIzol (Invitrogen) and total RNA was isolated as described above in mammalian cells. For each treatment, 3–4 μg of RNA was run on a northern blot that was probed with oligo c [5’-CAG GTA CCC GGG TCG GCC TGC GGC G-3’, [70]].

## Supporting information

S1 Fig siRNA depletion of PAX9 in multiple cell lines leads to pre-18S rRNA processing defects. (A) qRT-PCR confirmation of PAX9 siRNA knockdown in MCF10A, HEK294FT, and RKO cells. 2^^-ΔΔCt^ values, relative to a siNT control and 7SL control primer, show knockdown of PAX9 by qRT-PCR using the indicated siRNAs. Data are shown as mean ± SEM. Analysis was completed by Student’s t-test using GraphPad Prism where *** p ≤ 0.001 and **** p ≤ 0.0001. (B) Western blot showing depletion of PAX9 in MCF10A cells. Left: Representative western blots using antibodies for PAX9 or vinculin as a loading control. Mock and non-targeting (siNT) siRNAs are shown as negative controls. Right: Quantitation of PAX9 levels in 3 such western blots, relative to the siNT control and to the vinculin loading control. Analysis was completed using One-way ANOVA with Dunnett’s multiple comparisons test in GraphPad Prism where *** p ≤ 0.001 and * p ≤ 0.05. (C) Northern blot showing depletion of PAX9 in HEK294FT and RKO cells using probe P3. A probe for the 7SL RNA was used as a loading control. Mock and siNT were used as negative controls. PTP indicates the 47S, 45S, and 43S processing intermediates. (D) Ratio analysis of multiple precursors (RAMP, [40]) data for the P3 northern blot shown in (B). N = 3. Data are shown as mean ± SEM. Significance was calculated using 2-way ANOVA in GraphPad Prism. **** p ≤ 0.0001, *** p ≤ 0.001, and ** p ≤ 0.01. (E) Quantitation of the northern blot shown in (B) relative to a 7SL loading control. N = 3. Data are shown as mean ± SEM. Significance was calculated using 2-way ANOVA in GraphPad Prism. **** p ≤ 0.0001, *** p ≤ 0.001, and ** p ≤ 0.01.
(TIF)

S2 Fig Additional northern blots reveal small subunit (SSU) pre-rRNA processing defects after PAX9 depletion. (A) Schematic of the human 47S pre-rRNA with cleavage sites indicated above. Black boxes below the pre-rRNA indicate the northern blot probes used to examine PAX9’s role in pre-rRNA processing. (B) Left: Northern blot with 5’ETS probe. A probe for the 7SL RNA was used as a loading control. Intermediates detected by the 5’ETS probe are shown to the right of the northern blot. Right: Quantitation for RAMP of the 5’ETS probe (left) and 7SL (right) northern blots. Graph is mean ± SEM. N = 3. Data were analyzed by 2-way ANOVA using GraphPad Prism. PTP indicates the 47S, 45S, and 43S processing intermediates. (C) Northern blot with the P1 probe. Data shown as in (B). (D) Northern blot with the P2 probe. Data shown as in (B). (E) Northern blot with the 5’ITS1 probe. Data shown as in (B). (F) Northern blot with the P4 probe. Data shown as in (B).
(TIF)

S3 Fig Cell cycle analysis upon PAX9 siRNA knockdown in MCF10A cells. (A) Flow cytometry cell cycle analysis using propidium iodide staining on human MCF10A cells. One representative plot is shown for each of the siNT, siNOL11, and siPAX9 treatments. Cells were stained with propidium iodide after 72 hours knockdown with the indicated siRNAs. Live cells were analyzed by FACS and the percentage of cells in G1 (blue), S (yellow), or G2 (green) phase was quantified as indicated. (B) Quantitation of 3 different flow experiments using cells of different passage numbers. Data were analyzed by 2-way ANOVA using GraphPad Prism where * p ≤ 0.05.
(TIF)

S4 Fig siRNA depletion of PAX9 affects Wnt signaling in MCF10A cells. (A) The mRNAs with decreased expression upon PAX9 depletion are enriched for genes that influence the cell cycle and protein synthesis (left). The mRNAs with increased expression upon PAX9 depletion are enriched for genes that influence cell death and survival (right). Ingenuity Pathways Analysis (IPA; QIAGEN Inc., https://www.qiagenbioinformatics.com/products/ingenuitypathway-analysis) reveals Molecular and Cellular Functions that are enriched in the list of mRNAs with either decreased (left) or increased (right) expression upon PAX9 knockdown (S1 Table). Only pathways enriched with a -log(p-value), which measures the enrichment of the pathway in the RNA-seq dataset, of ≥ 5 are shown. (B) Schematic of the Wnt/Ca2+ signaling pathway. Pathway members differentially regulated (fold change ≥ 2 or ≤ -2 and FDR ≤ 0.05) after PAX9 knockdown in the RNA-seq analysis are highlighted in purple. Figure generated using IPA software [53]. (C) Schematic of the Wnt/β-catenin signaling pathway. Pathway members differentially regulated (fold change ≥ 2 or ≤ -2 and FDR ≤ 0.05) in the RNA-seq analysis after PAX9 knockdown are highlighted in purple. Figure generated using IPA software [53].
(TIF)

S5 Fig Quantitation of three replicates of the northern blots relative to the 7SL loading control reveals pre-rRNA processing defects after depletion of 4/5 RNA-seq tested candidates in MCF10A cells. Quantitation of the northern blot ratio of each intermediate detected by probe P3 relative to the 7SL loading control for the 5 RNA-seq candidates shown in Fig 2D. Data were normalized to the siNT control. N = 3. Data are plotted as mean ± SEM on a LOG_2_ scale. Statistical analysis was completed by 2-way ANOVA in GraphPad Prism where **** p ≤ 0.0001, *** p ≤ 0.001, and ** p ≤ 0.01.
(TIF)

S6 Fig Quantitation of three replicates of the northern blots relative to the 7SL loading control after depletion of 6 RNAPII ChIP-seq/RNA-seq candidates. Quantitation of the northern blot ratio of each intermediate detected by probe P3 relative to the 7SL loading control for the 6 RNAPII ChIP-seq/RNA-seq candidates shown in Fig 3E. Data are normalized to the siNT control. N = 3. Data are plotted as mean ± SEM on a LOG_2_ scale. Statistical analysis was completed by 2-way ANOVA in GraphPad Prism where **** p ≤ 0.0001, *** p ≤ 0.001, and ** p ≤ 0.01.
(TIF)

S7 Fig qRT-PCR after depletion of the indicated RNAPII ChIP-seq/RNA-seq candidates relative to the siNT control in MCF10A cells. After depletion of each hit using siRNAs for 72 hours, qRT-PCR was performed using primers targeting that gene of interest. Data are shown as 2^^-ΔΔCt^, relative to the siNT control. Three replicates using MCF10A cells of different passage numbers, each with three technical replicates, were performed for each qRT-PCR experiment. Data are shown as mean ± SEM. Analysis was completed using One-way ANOVA using GraphPad Prism where **** p ≤ 0.0001.
(TIF)

S8 Fig PAX9 depletion by both MO and CRISPR-mediated knockdown results in dysmorphic cartilogenesis in developing *X*. *tropicalis* embryos. Above: Quantitation of the percent of normal (blue) and facially deformed embryos (orange) at stage 45. Control and F0 PAX9 CRISPR embryos (sg#1 and sg#2) are shown on the left, and control and PAX9 MO treated embryos (15 and 20 ng MO) are shown on the right. The table underneath the graph conveys the numerical percentages for each condition. Embryos that did not survive to the evaluation stage were not included. Experiments in which the total death rate was higher than 15% were rejected in both MO or CRISPR knockdowns. Below: Inference of CRISPR Edits (ICE) analysis for the CRISPR sgRNAs #1 and #2. ICE score showing the editing efficiency percentage, knockout (KO) scores showing the proportion of cells with either a frameshift or indel that are likely to generate loss-of-function mutations, the mean discord, and sgRNA guide sequences are presented for each of the sgRNAs.
(TIF)

S9 Fig Quantification of reduced expression of Sox9, Twist, and Slug neural crest cell markers after *pax9* depletion shown in Fig 6B. The expression level of Sox9, Twist, or Slug mRNA for each embryo was categorized as either equal (blue) or reduced (orange) bilaterally, on the right side, or on the left side. For embryos depleted of *pax9* by MO injection on either the right or left side at the 2-cell stage, SOX9 expression was reduced by 30% (Right) and 25% (Left), Twist expression was reduced 32% (Right) and 29% (Left) and Slug expression was reduced by 37% (Right) and 35% (Left), respectively. UIC is the uninjected control.
(TIF)

S10 Fig Overexpression of human PAX9 mRNA alone in *X*. *tropicalis* embryos results in reduced Slug expression. Quantitation of the experiment shown in Fig 6D. At the 2-cell stage, human PAX9 mRNA was injected into 1 cell of the *X*. *tropicalis* embryos, with the other cell serving as an uninjected control (UIC). Slug expression was analyzed at stage 18 and was categorized as either equal (blue) or reduced (orange) bilaterally, on the right side, or on the left side. Controls showed 90% equal expression and 10% bilateral reduced expression. Left-side human PAX9 mRNA injection led to a reduction in Slug expression of 74% (Right-side injection) and 83% (Left-side injection).
(TIF)

S11 Fig p53 levels are increased when Pax9 is depleted. (A) Left: p53 levels are increased when PAX9 is depleted by siRNA in RKO cells. Western blot with an antibody to p53 or to a β actin loading control. Protein was harvested from mammalian RKO cells depleted using siRNAs targeting Mock (negative control), non-targeting (NT, negative control), NOL11 (positive control), or PAX9. Right: Quantitation of 6 replicates of the western blots using cells of different passage numbers. Data are shown as mean ± SEM. Significance was calculated by Student’s t-test using GraphPad Prism where * p ≤ 0.05. (B) Left: p53 levels are increased when *pax9* is depleted in the developing *Xenopus tropicalis* embryo. Representative western blot with an antibody to p53 or a GAPDH loading control. Protein was harvested from *X*. *tropicalis* embryos depleted of *pax9* using 2 non-overlapping CRISPR sgRNAs, a MO targeting Pax9, or an uninjected control (UIC). Right: Data are shown as mean ± SEM. N = 3 for all except sg#2 stage 25 and MO stage 46 where N = 2. Significance was calculated by Student’s t-test using GraphPad Prism where ** p ≤ 0.01 and * p ≤ 0.05.
(TIF)

S1 Table RNA-seq analysis of PAX9-depleted MCF10A cells relative to siNT control. Tab 1. Cufflinks [89] results from the RNA-seq analysis. See Materials and Methods for further details. Tab 2. Comparison of the RNA-seq hits to genome-wide siRNA screens for ribosome biogenesis factors (Farley-Barnes et al [34], Badertscher et al [57], and Neumüller Yeast and Neumüller *Drosophila* referring to the siRNA screens in yeast and Drosophila, respectively [58]). A 1 denotes a mRNA that was included as a hit in that siRNA screen, while a 0 indicates that the mRNA was not considered a hit in that screen. For the second column, a 1 denotes that the mRNA had decreased expression (fold change ≤ - 2) in the RNA-seq dataset, while a 0 indicates denotes that the mRNA had increased expression (fold change ≥ - 2) in the RNA-seq dataset. Tab 3. Comparison of all RNA-seq differentially expressed mRNAs to 3 databases of nucleolar proteins: the Human Protein Atlas [52], the NOPdb [51], and nucleolar proteins in human T-cells from the Gautier laboratory [50]. A 1 denotes a mRNA that was included in the indicated database, while a 0 indicates that the mRNA was not included in the indicated database. For the second column, a 1 denotes that the mRNA had decreased expression (fold change ≤ - 2) in the RNA-seq dataset, while a 0 indicates denotes that the mRNA had increased expression (fold change ≥ - 2) in the RNA-seq dataset. Tab 4. Comparison of all RNA-seq differentially expressed mRNAs to genes differentially occupied by PAX9 in the vertebral columns of E12.5 mice by PAX9 ChIP-seq (Sivakamasundari et al. [54]). A 1 denotes a mRNA that was regulated in the ChIP-seq experiment, while a 0 indicates that the mRNA was not regulated in the ChIP-seq experiment. For the second column, a 1 denotes that the mRNA had decreased expression (fold change ≤ - 2) in the RNA-seq dataset, while a 0 indicates denotes that the mRNA had increased expression (fold change ≥ - 2) in the RNA-seq dataset.
(XLSX)

S2 Table Dharmacon catalog numbers (siGENOME) for each siRNA used in this study. (DOCX)

S3 Table List of primers used in this study. For each target, the forward (F primer) and reverse (R primer) sequences are listed in the 5’-3’ direction. Where applicable, the PrimerBank ID number [96] or citation [97–102] is also listed. N/a indicates primers designed for this study that were not previously published or included in the PrimerBank database.
(DOCX)

S4 Table Data underlying graphs and summary statistics. Data is presented as a spreadsheet with one tab for each main figure and supplemental figure.
(XLSX)

S1 Movie Optical Coherence Tomography (OCT) imaging of uninjected control (CONTROL, left) vs CRISPR-mediated *pax9* knockout (Pax9 F0 mutant, right) *X*. *tropicalis* embryos. Branchial (gill), ceratohyal, and Meckel’s cartilages are indicated in both embryos.
(MP4)
