# Supplementary figures and images for: Paired Box 9 (PAX9), the RNA polymerase II transcription factor, regulates human ribosome biogenesis and craniofacial development
Source: PLoS Genet. 2020 Aug 19;16(8):e1008967. doi: 10.1371/journal.pgen.1008967 (PMC7437866; doi:10.1371/journal.pgen.1008967)

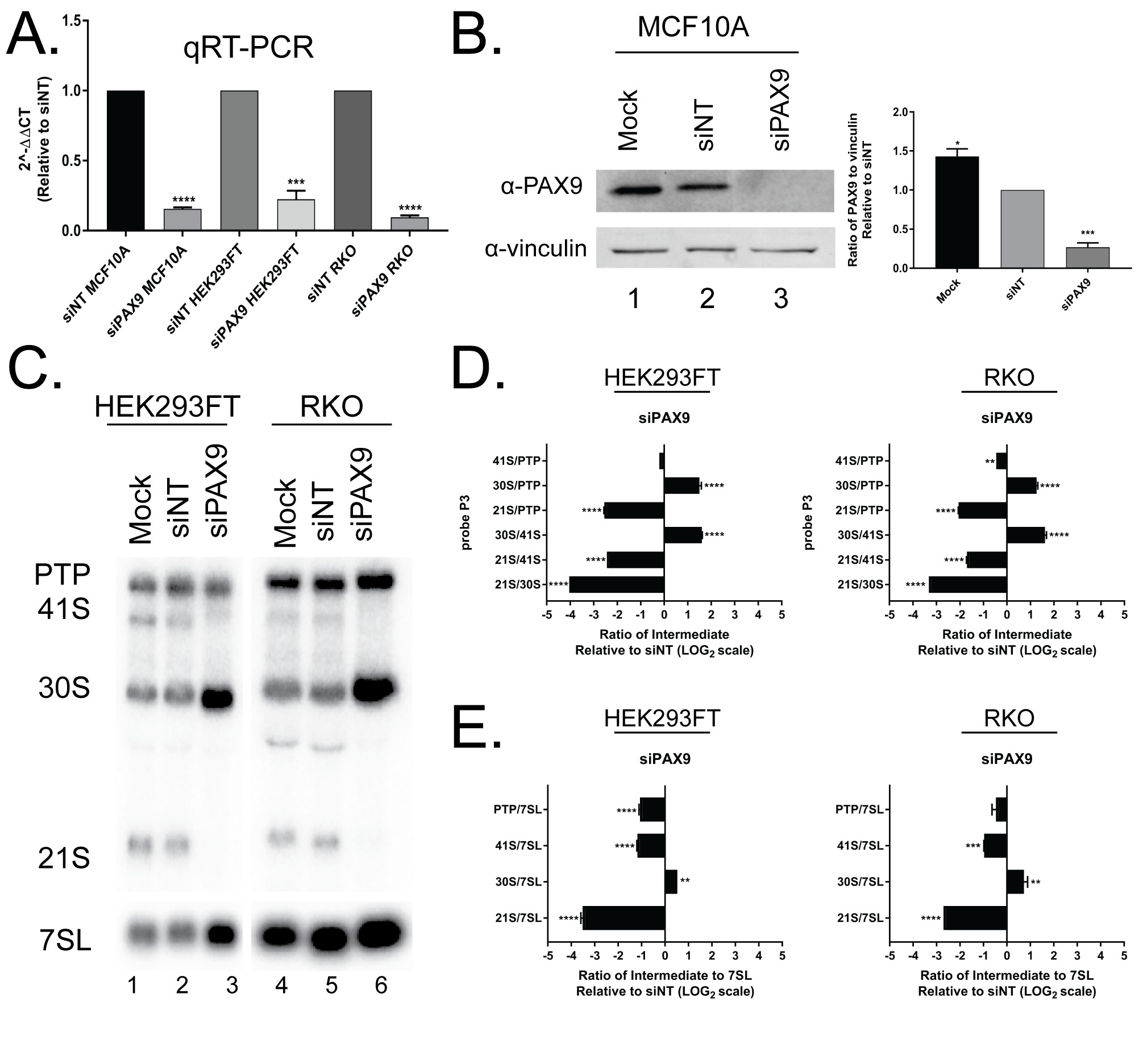

Supplement: S1 Fig — (A) qRT-PCR confirmation of PAX9 siRNA knockdown in MCF10A, HEK294FT, and RKO cells. 2^-ΔΔCt values, relative to a siNT control and 7SL control primer, show knockdown of PAX9 by qRT-PCR using the indicated siRNAs. Data are shown as mean ± SEM. Analysis was completed by Student’s t-test using GraphPad Prism where *** p ≤ 0.001 and **** p ≤ 0.0001. (B) Western blot showing depletion of PAX9 in MCF10A cells. Left: Representative western blots using antibodies for PAX9 or vinculin as a loading control. Mock and non-targeting (siNT) siRNAs are shown as negative controls. Right: Quantitation of PAX9 levels in 3 such western blots, relative to the siNT control and to the vinculin loading control. Analysis was completed using One-way ANOVA with Dunnett’s multiple comparisons test in GraphPad Prism where *** p ≤ 0.001 and * p ≤ 0.05. (C) Northern blot showing depletion of PAX9 in HEK294FT and RKO cells using probe P3. A probe for the 7SL RNA was used as a loading control. Mock and siNT were used as negative controls. PTP indicates the 47S, 45S, and 43S processing intermediates. (D) Ratio analysis of multiple precursors (RAMP, [40]) data for the P3 northern blot shown in (B). N = 3. Data are shown as mean ± SEM. Significance was calculated using 2-way ANOVA in GraphPad Prism. **** p ≤ 0.0001, *** p ≤ 0.001, and ** p ≤ 0.01. (E) Quantitation of the northern blot shown in (B) relative to a 7SL loading control. N = 3. Data are shown as mean ± SEM. Significance was calculated using 2-way ANOVA in GraphPad Prism. **** p ≤ 0.0001, *** p ≤ 0.001, and ** p ≤ 0.01. (TIF) [file pgen.1008967.s001.tif]

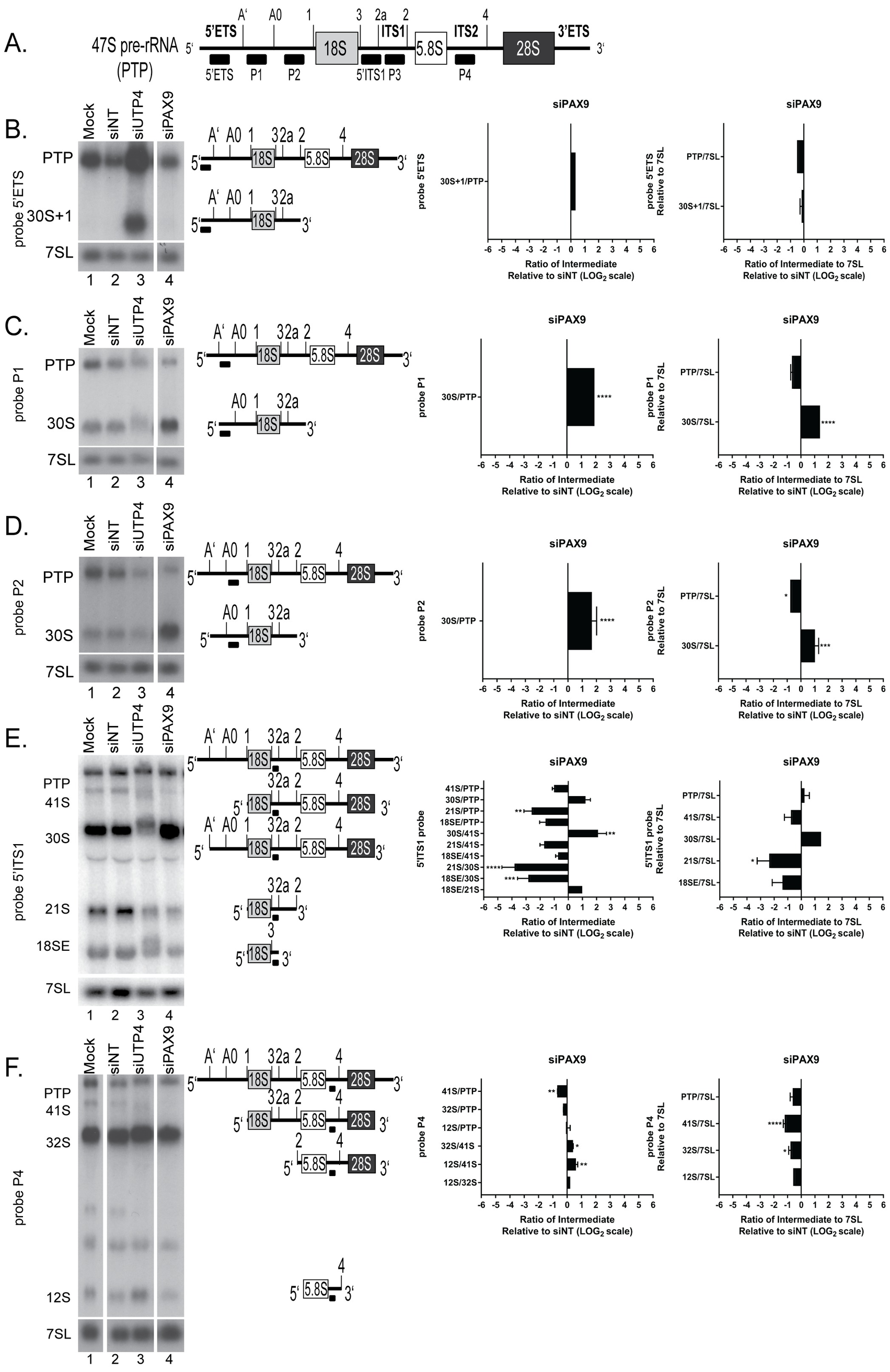

Supplement: S2 Fig — (A) Schematic of the human 47S pre-rRNA with cleavage sites indicated above. Black boxes below the pre-rRNA indicate the northern blot probes used to examine PAX9’s role in pre-rRNA processing. (B) Left: Northern blot with 5’ETS probe. A probe for the 7SL RNA was used as a loading control. Intermediates detected by the 5’ETS probe are shown to the right of the northern blot. Right: Quantitation for RAMP of the 5’ETS probe (left) and 7SL (right) northern blots. Graph is mean ± SEM. N = 3. Data were analyzed by 2-way ANOVA using GraphPad Prism. PTP indicates the 47S, 45S, and 43S processing intermediates. (C) Northern blot with the P1 probe. Data shown as in (B). (D) Northern blot with the P2 probe. Data shown as in (B). (E) Northern blot with the 5’ITS1 probe. Data shown as in (B). (F) Northern blot with the P4 probe. Data shown as in (B). (TIF) [file pgen.1008967.s002.tif]

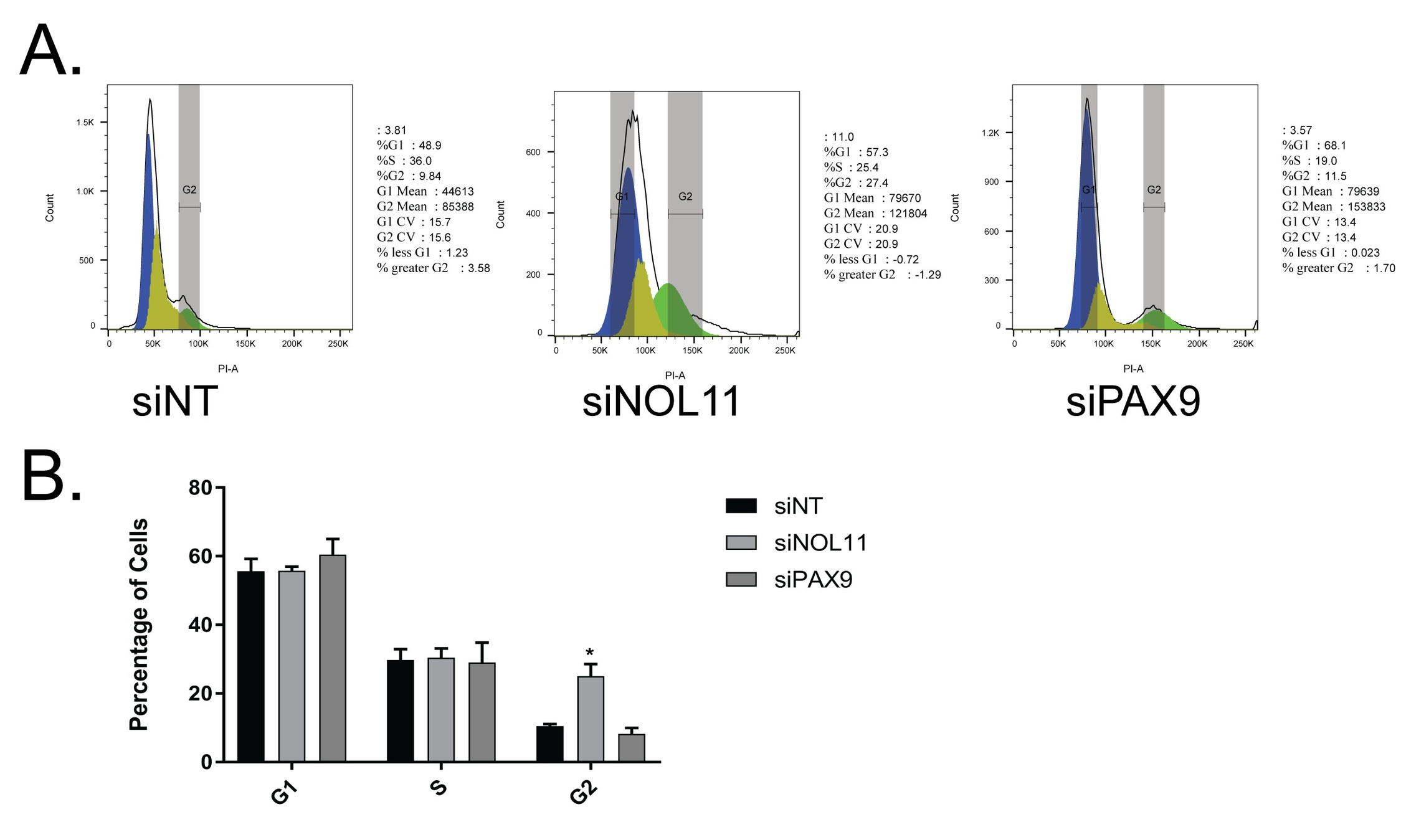

Supplement: S3 Fig — (A) Flow cytometry cell cycle analysis using propidium iodide staining on human MCF10A cells. One representative plot is shown for each of the siNT, siNOL11, and siPAX9 treatments. Cells were stained with propidium iodide after 72 hours knockdown with the indicated siRNAs. Live cells were analyzed by FACS and the percentage of cells in G1 (blue), S (yellow), or G2 (green) phase was quantified as indicated. (B) Quantitation of 3 different flow experiments using cells of different passage numbers. Data were analyzed by 2-way ANOVA using GraphPad Prism where * p ≤ 0.05. (TIF) [file pgen.1008967.s003.tif]

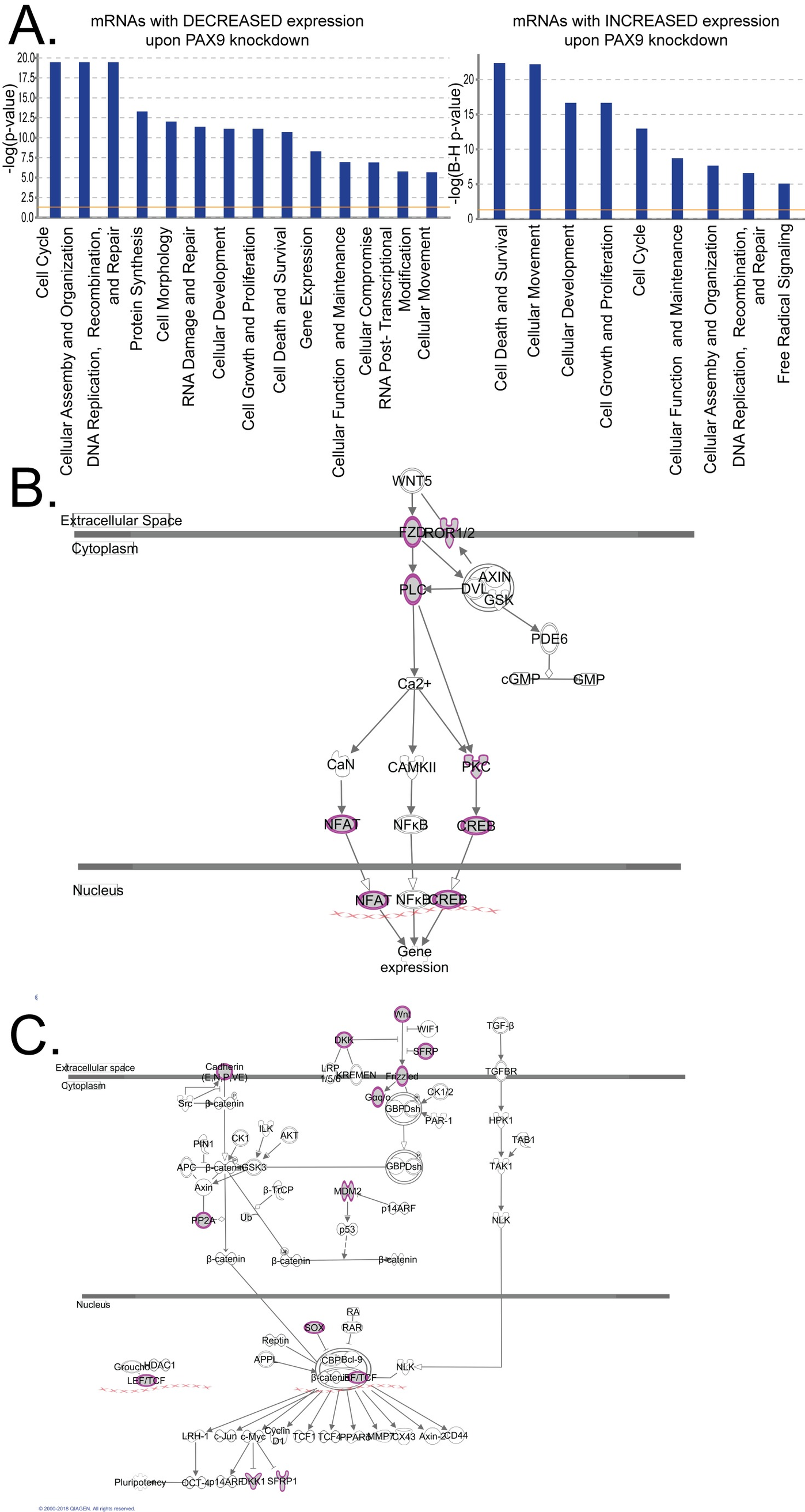

Supplement: S4 Fig — (A) The mRNAs with decreased expression upon PAX9 depletion are enriched for genes that influence the cell cycle and protein synthesis (left). The mRNAs with increased expression upon PAX9 depletion are enriched for genes that influence cell death and survival (right). Ingenuity Pathways Analysis (IPA; QIAGEN Inc., https://www.qiagenbioinformatics.com/products/ingenuitypathway-analysis) reveals Molecular and Cellular Functions that are enriched in the list of mRNAs with either decreased (left) or increased (right) expression upon PAX9 knockdown (S1 Table). Only pathways enriched with a -log(p-value), which measures the enrichment of the pathway in the RNA-seq dataset, of ≥ 5 are shown. (B) Schematic of the Wnt/Ca2+ signaling pathway. Pathway members differentially regulated (fold change ≥ 2 or ≤ -2 and FDR ≤ 0.05) after PAX9 knockdown in the RNA-seq analysis are highlighted in purple. Figure generated using IPA software [53]. (C) Schematic of the Wnt/β-catenin signaling pathway. Pathway members differentially regulated (fold change ≥ 2 or ≤ -2 and FDR ≤ 0.05) in the RNA-seq analysis after PAX9 knockdown are highlighted in purple. Figure generated using IPA software [53]. (TIF) [file pgen.1008967.s004.tif]

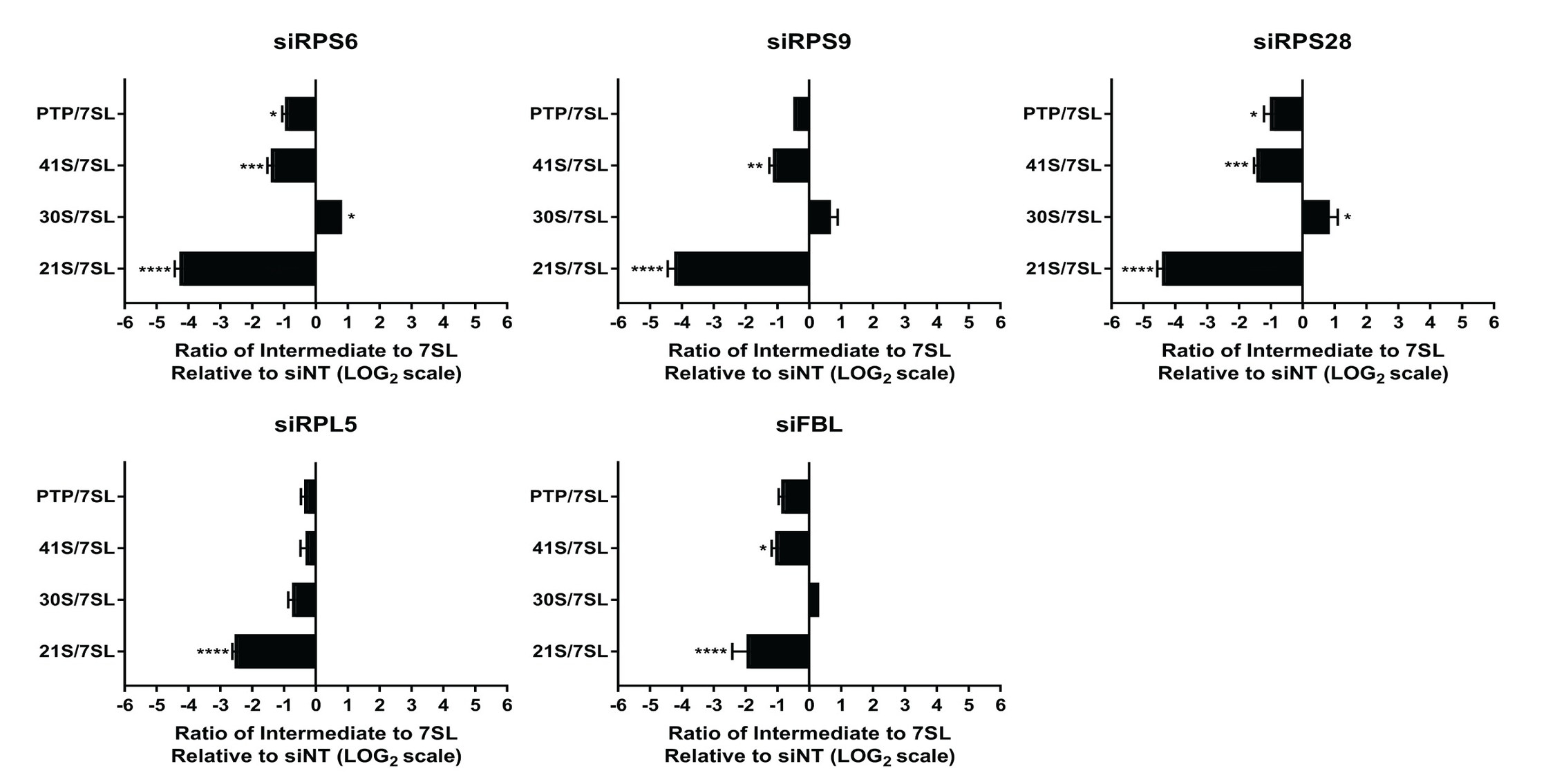

Supplement: S5 Fig — Quantitation of the northern blot ratio of each intermediate detected by probe P3 relative to the 7SL loading control for the 5 RNA-seq candidates shown in Fig 2D. Data were normalized to the siNT control. N = 3. Data are plotted as mean ± SEM on a LOG2 scale. Statistical analysis was completed by 2-way ANOVA in GraphPad Prism where **** p ≤ 0.0001, *** p ≤ 0.001, and ** p ≤ 0.01. (TIF) [file pgen.1008967.s005.tif]

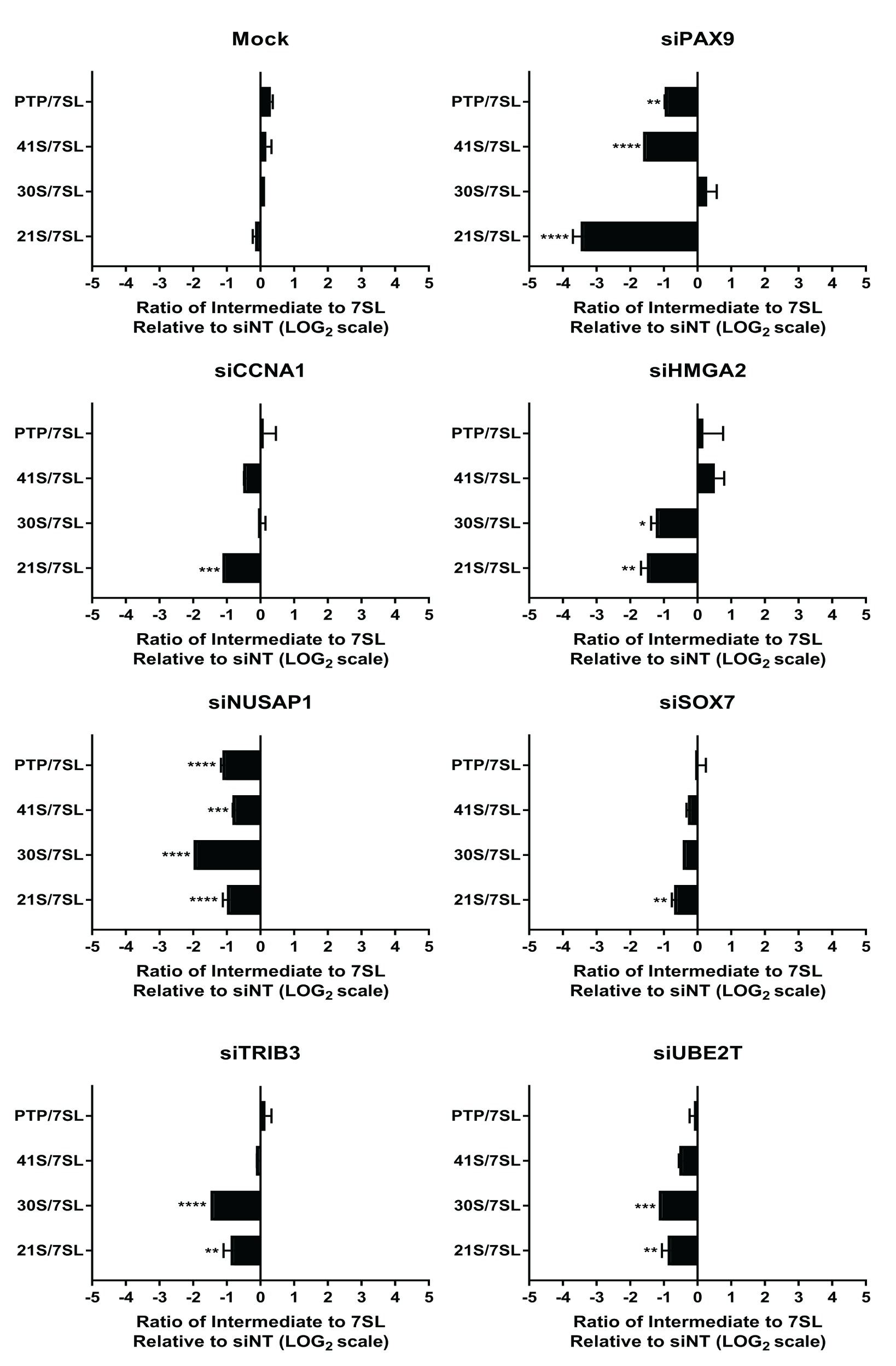

Supplement: S6 Fig — Quantitation of the northern blot ratio of each intermediate detected by probe P3 relative to the 7SL loading control for the 6 RNAPII ChIP-seq/RNA-seq candidates shown in Fig 3E. Data are normalized to the siNT control. N = 3. Data are plotted as mean ± SEM on a LOG2 scale. Statistical analysis was completed by 2-way ANOVA in GraphPad Prism where **** p ≤ 0.0001, *** p ≤ 0.001, and ** p ≤ 0.01. (TIF) [file pgen.1008967.s006.tif]

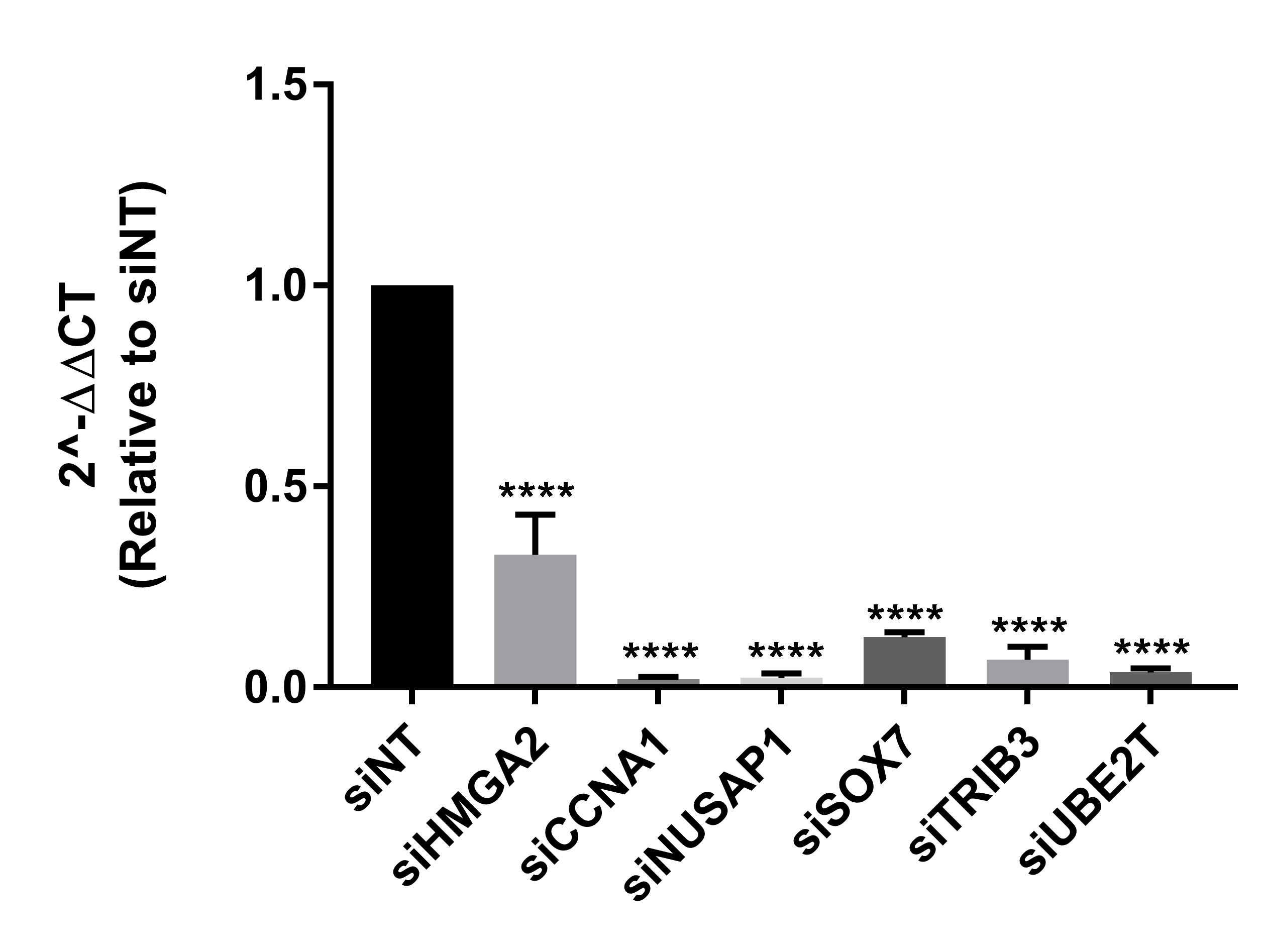

Supplement: S7 Fig — After depletion of each hit using siRNAs for 72 hours, qRT-PCR was performed using primers targeting that gene of interest. Data are shown as 2^-ΔΔCt, relative to the siNT control. Three replicates using MCF10A cells of different passage numbers, each with three technical replicates, were performed for each qRT-PCR experiment. Data are shown as mean ± SEM. Analysis was completed using One-way ANOVA using GraphPad Prism where **** p ≤ 0.0001. (TIF) [file pgen.1008967.s007.tif]

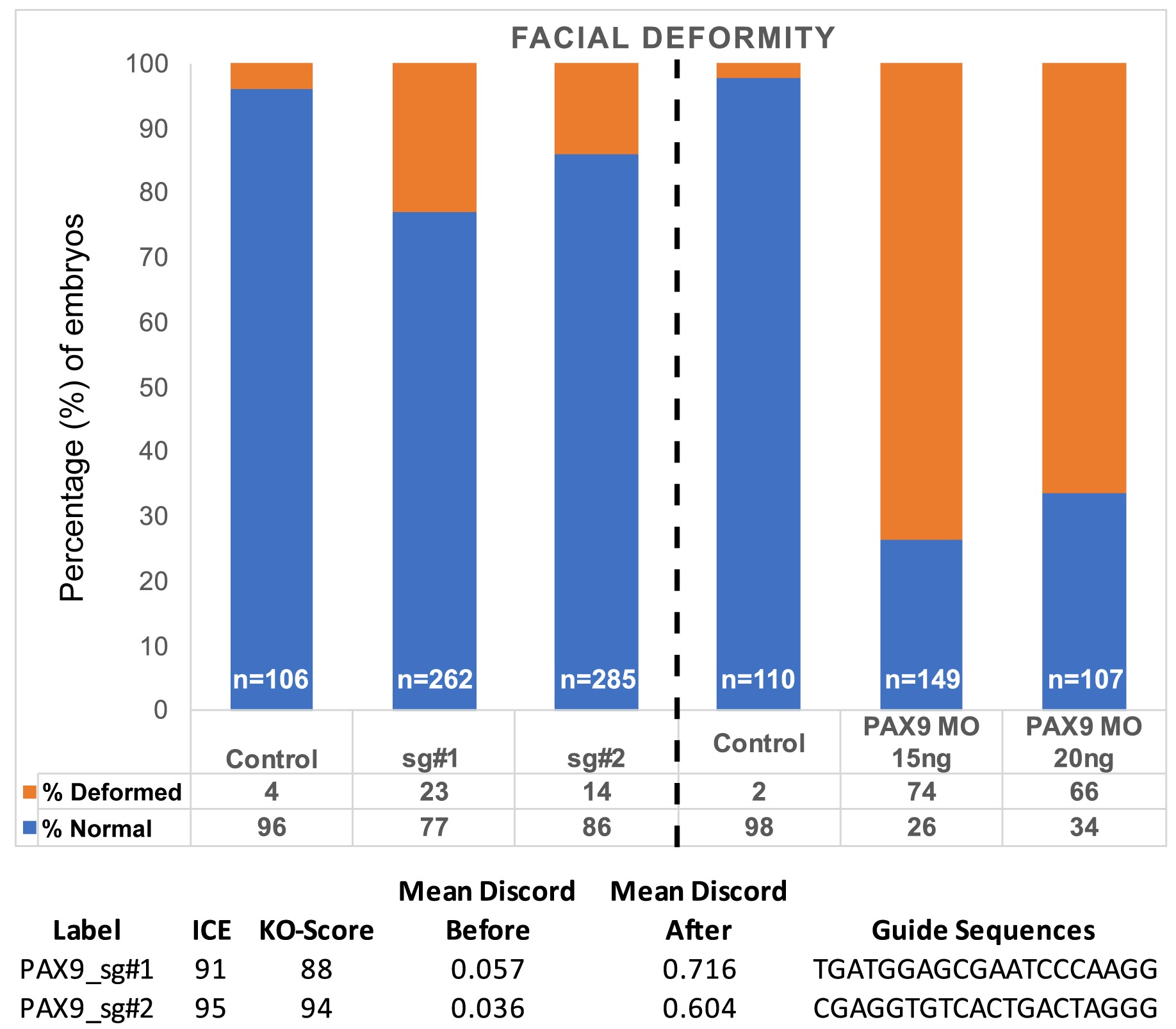

Supplement: S8 Fig — Above: Quantitation of the percent of normal (blue) and facially deformed embryos (orange) at stage 45. Control and F0 PAX9 CRISPR embryos (sg#1 and sg#2) are shown on the left, and control and PAX9 MO treated embryos (15 and 20 ng MO) are shown on the right. The table underneath the graph conveys the numerical percentages for each condition. Embryos that did not survive to the evaluation stage were not included. Experiments in which the total death rate was higher than 15% were rejected in both MO or CRISPR knockdowns. Below: Inference of CRISPR Edits (ICE) analysis for the CRISPR sgRNAs #1 and #2. ICE score showing the editing efficiency percentage, knockout (KO) scores showing the proportion of cells with either a frameshift or indel that are likely to generate loss-of-function mutations, the mean discord, and sgRNA guide sequences are presented for each of the sgRNAs. (TIF) [file pgen.1008967.s008.tif]

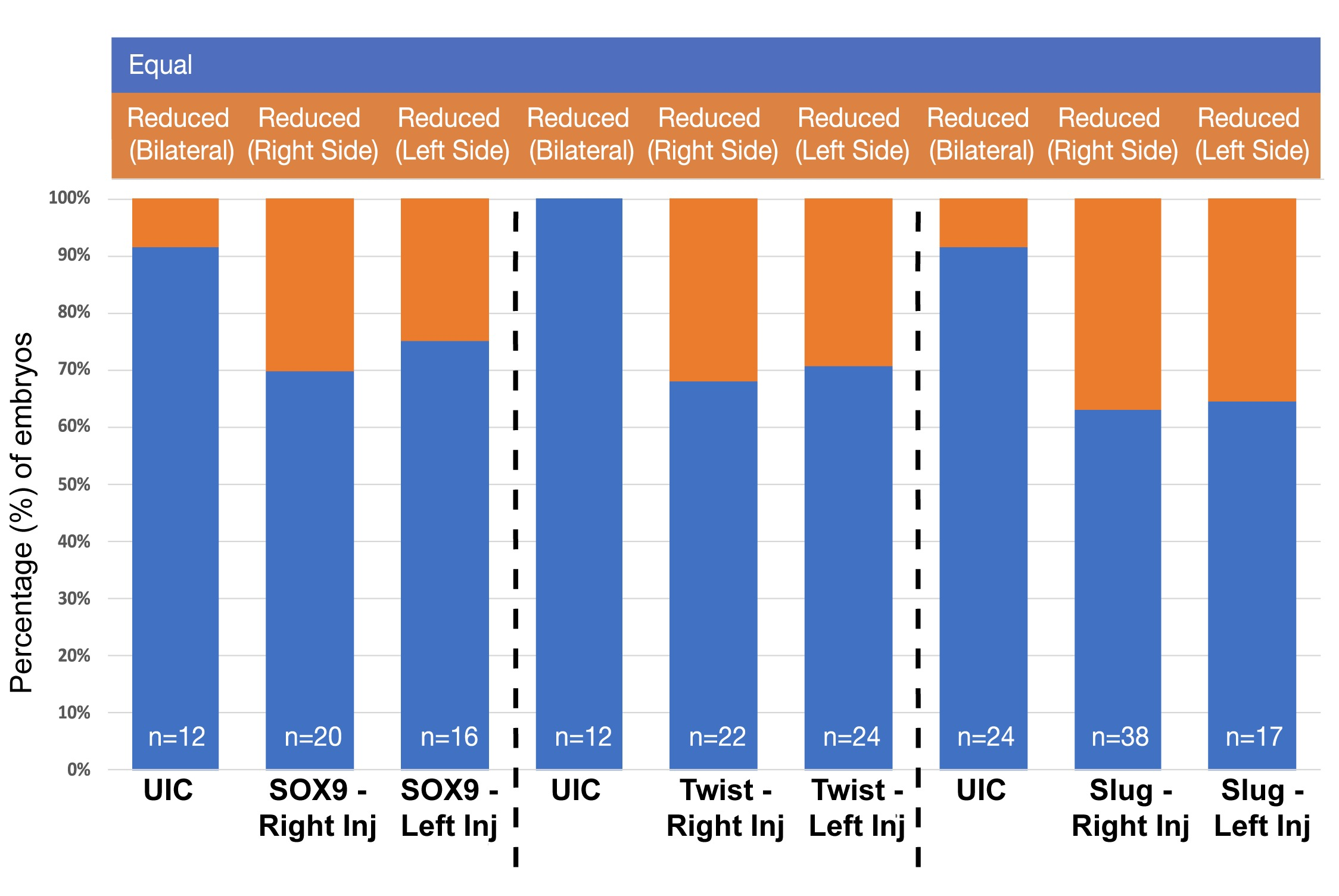

Supplement: S9 Fig — The expression level of Sox9, Twist, or Slug mRNA for each embryo was categorized as either equal (blue) or reduced (orange) bilaterally, on the right side, or on the left side. For embryos depleted of pax9 by MO injection on either the right or left side at the 2-cell stage, SOX9 expression was reduced by 30% (Right) and 25% (Left), Twist expression was reduced 32% (Right) and 29% (Left) and Slug expression was reduced by 37% (Right) and 35% (Left), respectively. UIC is the uninjected control. (TIF) [file pgen.1008967.s009.tif]

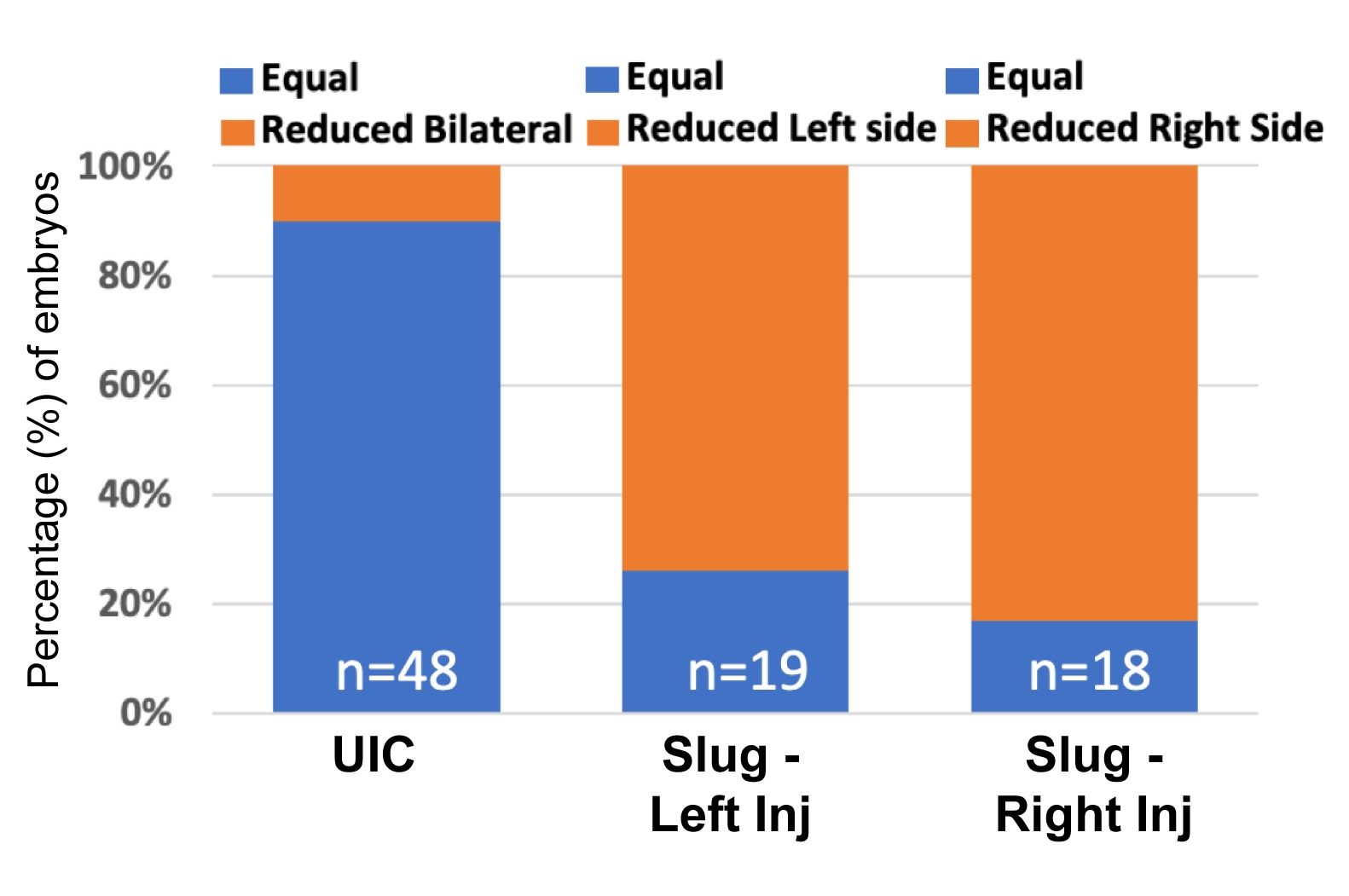

Supplement: S10 Fig — Quantitation of the experiment shown in Fig 6D. At the 2-cell stage, human PAX9 mRNA was injected into 1 cell of the X. tropicalis embryos, with the other cell serving as an uninjected control (UIC). Slug expression was analyzed at stage 18 and was categorized as either equal (blue) or reduced (orange) bilaterally, on the right side, or on the left side. Controls showed 90% equal expression and 10% bilateral reduced expression. Left-side human PAX9 mRNA injection led to a reduction in Slug expression of 74% (Right-side injection) and 83% (Left-side injection). (TIF) [file pgen.1008967.s010.tif]

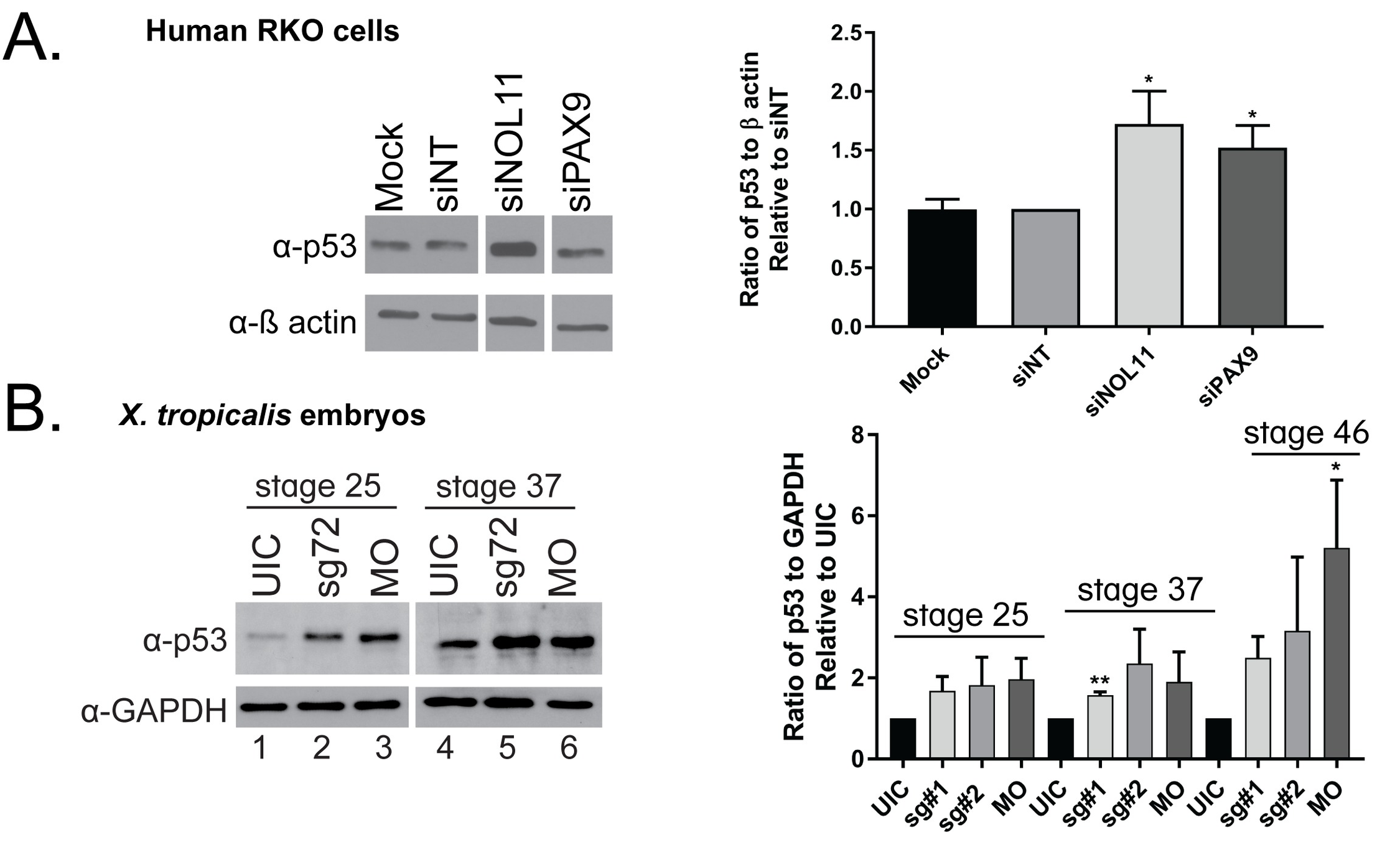

Supplement: S11 Fig — (A) Left: p53 levels are increased when PAX9 is depleted by siRNA in RKO cells. Western blot with an antibody to p53 or to a β actin loading control. Protein was harvested from mammalian RKO cells depleted using siRNAs targeting Mock (negative control), non-targeting (NT, negative control), NOL11 (positive control), or PAX9. Right: Quantitation of 6 replicates of the western blots using cells of different passage numbers. Data are shown as mean ± SEM. Significance was calculated by Student’s t-test using GraphPad Prism where * p ≤ 0.05. (B) Left: p53 levels are increased when pax9 is depleted in the developing Xenopus tropicalis embryo. Representative western blot with an antibody to p53 or a GAPDH loading control. Protein was harvested from X. tropicalis embryos depleted of pax9 using 2 non-overlapping CRISPR sgRNAs, a MO targeting Pax9, or an uninjected control (UIC). Right: Data are shown as mean ± SEM. N = 3 for all except sg#2 stage 25 and MO stage 46 where N = 2. Significance was calculated by Student’s t-test using GraphPad Prism where ** p ≤ 0.01 and * p ≤ 0.05. (TIF) [file pgen.1008967.s011.tif]
