# Supplementary material for: Paired Box 9 (PAX9), the RNA polymerase II transcription factor, regulates human ribosome biogenesis and craniofacial development
Source: PLoS Genet. 2020 Aug 19;16(8):e1008967. doi: 10.1371/journal.pgen.1008967 (PMC7437866; doi:10.1371/journal.pgen.1008967)
Supplement: S2 Table — (DOCX) [file pgen.1008967.s013.docx]

| siRNA target | Catalog Number |
| --- | --- |
| siCCNA1 | M-003204-02-0005 |
| siFBL | M-011269-00-0005 |
| siHMGA2 | M-013495-02-0005 |
| siNOL11 | M-016695-01-0005 |
| siNT (non-targeting) | D-001810-10-20 |
| siNUSAP1 | M-004754-04-0005 |
| siPAX1 | M-020120-01-0005 |
| siPAX9 | M-012242-01-0005 |
| siRPL5 | M-013611-01-0005 |
| siRPS28 | M-013679-01-0005 |
| siRPS6 | M-003204-01-0005 |
| siRPS9 | M-011131-01-0005 |
| siSOX7 | M-019017-00-0005 |
| siTRIB3 | M-003754-01-0005 |
| siUBE2T | M-004898-01-0005 |
| siUTP4 | M-015011-01-0005 |
