# Supplementary material for: Paired Box 9 (PAX9), the RNA polymerase II transcription factor, regulates human ribosome biogenesis and craniofacial development
Source: PLoS Genet. 2020 Aug 19;16(8):e1008967. doi: 10.1371/journal.pgen.1008967 (PMC7437866; doi:10.1371/journal.pgen.1008967)
Supplement: S3 Table — For each target, the forward (F primer) and reverse (R primer) sequences are listed in the 5’-3’ direction. Where applicable, the PrimerBank ID number [96] or citation [97–102] is also listed. N/a indicates primers designed for this study that were not previously published or included in the PrimerBank database. (DOCX) [file pgen.1008967.s014.docx]

| Target Gene Name | F primer (5’-3’) | R primer (5’-3’) | Citation or  PrimerBank ID |
| --- | --- | --- | --- |
| 7SL | ATCGGGTGTCCGCACTAAGT | CAGCACGGGAGTTTTGACCT | [97] |
| CCNA1 | TAGACACCGGCACACTCAAG | AGGAGAGATGAATCTACCAGCAT | 161377466c3 |
| FBL | CCTGCGTAATGGAGGACACT | ACTTCGGAGGCAAACACG | n/a |
| HMGA2 | CCCAAAGGCAGCAAAAACAA | GCCTCTTGGCCGTTTTTCTC | [98] |
| NUSAP1 | CCCTCAAGTACAGTGACCTGC | TCATTTCCTTTTCTTGCCTCA | [99] |
| PAX9 | GAAAGTTTCTGTCTGGGAGTGC | TCCCAGCTGGTTCACCTC | n/a |
| RPL5 | GCCCAAGAAAGAAGTTAAAAAGAAG | CATAGAAAATTGCTGGGTTTAGC | n/a |
| RPS28 | GGTCTGTCACAGTCTGCTCC | CATCTCAGTTACGTGTGGCG | [100] |
| RPS6 | CTGACGCTCTGGGTGAAGAA | AAACCTTGTTTGTCGTTCCCAC | n/a |
| RPS9 | GCGGAGACCCTTCGAGAAAT | CCTCCAGACCTCACGTTTGT | n/a |
| SOX7 | GCCAAGGACGAGAGGAAAC | GTTGGGGTAGTCCTGCATGT | [101] |
| TRIB3 | GCCTTTTTCACTCGGACCCAT | CAGCGAAGACAAAGCGACAC | 41327717c3 |
| UBE2T | TTGATTCTGCTGGAAGGATTTG | CAGTTGCGATGTTGAGGGAT | [102] |
